# Phenotypic Classification of Multisystem Inflammatory Syndrome in Children Using Latent Class Analysis

**DOI:** 10.1001/jamanetworkopen.2024.56272

**Published:** 2025-01-28

**Authors:** Kevin C. Ma, Anna R. Yousaf, Allison Miller, Katherine N. Lindsey, Michael J. Wu, Michael Melgar, Ami B. Popovic, Angela P. Campbell, Laura D. Zambrano

**Affiliations:** 1 Coronavirus and Other Respiratory Viruses Division, National Center for Immunization and Respiratory Diseases, Centers for Disease Control and Prevention, Atlanta, Georgia; 2 Epidemic Intelligence Service, Centers for Disease Control and Prevention, Atlanta, Georgia; 3 4ES Corporation, San Antonio, Texas; 4 General Dynamics Information Technology, Falls Church, Virginia

## Abstract

**Importance:**

Multisystem inflammatory syndrome in children (MIS-C) is an uncommon but severe hyperinflammatory illness that occurs 2 to 6 weeks after SARS-CoV-2 infection. Presentation overlaps with other conditions, and risk factors for severity differ by patient. Characterizing patterns of MIS-C presentation can guide efforts to reduce misclassification, categorize phenotypes, and identify patients at risk for severe outcomes.

**Objective:**

To characterize phenotypic clusters of MIS-C and identify clusters with increased clinical severity.

**Design, Setting, and Participants:**

In this cohort study, MIS-C phenotypic clusters were inferred using latent class analysis applied to the largest cohort to date of cases from US national surveillance data from 55 US public health jurisdictions. Cases reported to the Centers for Disease Control and Prevention MIS-C national surveillance program as of April 4, 2023, with symptom onset on or before December 31, 2022, were retrospectively analyzed. Twenty-nine clinical signs and symptoms were selected for clustering after excluding variables with 20% or more missingness and 10% or less or 90% or more prevalence. A total of 389 cases missing 10 or more variables were excluded, and multiple imputation was conducted on the remaining cases.

**Main Outcomes and Measures:**

Differences by cluster in prevalence of each clinical sign and symptom, percentage of patients admitted to the intensive care unit (ICU), length of hospital and ICU stay, mortality, and relative frequency over time.

**Results:**

Among 8944 included MIS-C cases (median [IQR] patient age, 8.7 [5.0-12.5] years; 5407 [60.5%] male), latent class analysis identified 3 clusters characterized by (1) frequent respiratory findings primarily affecting older children (respiratory cluster; 713 cases [8.0%]; median [IQR] age, 12.7 [6.3-16.5] years), (2) frequent shock and/or cardiac complications (shock and cardiac cluster; 3359 cases [37.6%]; median [IQR] age, 10.8 [7.7-14.0] years), and (3) remaining cases (undifferentiated cluster; 4872 cases [54.5%]; median [IQR] age, 6.8 [3.6-10.3] years). The percentage of patients with MIS-C admitted to the ICU was highest for the shock and cardiac cluster (82.3% [2765/3359]) followed by the respiratory (49.5% [353/713]) and undifferentiated clusters (33.0% [1609/4872]). Among patients with data on length of stay available, 129 of 632 hospitalizations (20.4%) and 54 of 281 ICU stays (19.2%) in the respiratory cluster lasted 10 or more days compared with 708 of 3085 (22.9%) and 157 of 2052 (7.7%), respectively, in the shock and cardiac cluster and 293 of 4467 (6.6%) and 19 of 1220 (1.6%), respectively, in the undifferentiated cluster. The proportion of cases in both the respiratory cluster and the shock and cardiac cluster decreased after emergence of the Omicron variant in the US.

**Conclusions and Relevance:**

In this cohort study, MIS-C cases clustered into 3 subgroups with distinct clinical phenotypes, severity, and distribution over time. Use of clusters in future studies may support efforts to evaluate surveillance case definitions and identify groups at highest risk for severe outcomes.

## Key Points

**Question:**

Can phenotypic clusters of multisystem inflammatory syndrome in children (MIS-C) be identified, and are some clusters associated with increased severity?

**Findings:**

In this cohort study, a latent class analysis was conducted on 8944 MIS-C cases identified from US national surveillance data from 2020 to 2022, identifying 3 clusters characterized by frequent respiratory symptoms, frequent shock and cardiac complications, and decreased clinical severity. Mortality and intensive care unit admission were highest in the respiratory cluster and shock and cardiac cluster; prevalence of these clusters decreased over time.

**Meaning:**

The MIS-C clusters had distinct presentation, severity, and distribution over time, highlighting the importance of recognizing the varied presentation of MIS-C.

## Teaser

This cohort study characterizes phenotypic clusters of multisystem inflammatory syndrome in children and investigates their association with clinical severity.

## Introduction

Multisystem inflammatory syndrome in children (MIS-C) is an uncommon but severe hyperinflammatory syndrome that occurs 2 to 6 weeks after SARS-CoV-2 infection, postulated to arise because of postinfectious immune dysregulation.^1,2^ As of December 2, 2024, there have been 9750 MIS-C cases and 80 associated deaths in the US reported to the Centers for Disease Control and Prevention (CDC) national surveillance system, with higher incidence among racial and ethnic minority children and youths early in the pandemic.^3,4,5^ Patients with MIS-C commonly present with fever; gastrointestinal symptoms, including vomiting, diarrhea, and abdominal pain; cardiac dysfunction; and mucocutaneous symptoms, including rash and conjunctivitis.^6^ In May 2020, the CDC developed a case definition for MIS-C using the following criteria: hospitalized patients younger than 21 years with fever, involvement of 2 or more organ systems, laboratory evidence of inflammation, laboratory or epidemiologic evidence of current or recent SARS-CoV-2 infection, and no alternative plausible diagnosis.^7,8^ This definition was broad to maximize MIS-C case capture. Effective January 2023, the CDC and the Council for State and Territorial Epidemiologists (CSTE) created a new MIS-C surveillance case definition to reduce misclassification and minimize reporting burden.^9^ Changes included removal of the respiratory organ involvement criterion to potentially decrease misclassification of severe acute pediatric COVID-19 as MIS-C.

MIS-C case finding potentially captures a range of conditions because presentation shares clinical features with other conditions, including acute COVID-19, Kawasaki disease, and toxic shock syndrome, which may complicate timely diagnosis and treatment.^6,10,11^ Clinical severity (including the occurrence of shock, intensive care unit [ICU] admission, and death) can also vary substantially,^12,13^ and understanding of risk factors for severe disease remains incomplete.

This heterogeneity in presentation and clinical severity suggests the existence of phenotypic classes of MIS-C, as has been documented for other conditions, including acute respiratory distress syndrome.^14^ Characterizing these classes could improve understanding of MIS-C pathophysiology, guide subgroup analyses in clinical trials of therapeutic options, and support refinement of case definitions and epidemiologic surveillance. Previous studies have used latent class analysis (LCA) and other algorithms to characterize the spectrum of MIS-C presentation.^7,10,15^ These analyses have informed MIS-C case definition updates and highlighted how statistical models can help interpret MIS-C heterogeneity^9,16^ but have been limited in sample size and geographic representativeness. Additionally, some prior studies^7,15^ have aggregated input clinical data into broad organ system categories, which loses information on both severity and number of symptoms affecting organ systems.

The landscape of MIS-C incidence, surveillance, and treatment has changed considerably since SARS-CoV-2 first emerged, highlighting the need for an updated categorization of MIS-C phenotypic clusters. Overall incidence of MIS-C has decreased as new SARS-CoV-2 variants have emerged,^17,18,19,20,21,22^ but increases in MIS-C cases can still occur after surges in COVID-19 transmission.^23,24^ In this study, we apply statistical clustering methods to the largest cohort of US MIS-C cases to date to systematically characterize MIS-C phenotypic clusters and investigate their association with clinical severity.

## Methods

### Study Design and Cohort

The CDC started national passive surveillance for MIS-C cases on May 14, 2020. US health departments voluntarily reported cases using a standardized case report form that included questions on patient demographics, clinical signs and symptoms, treatments, and imaging and laboratory results (eAppendix in Supplement 1).^25^ Submitted case report forms were reviewed through automated and manual data checks for completeness and to ensure all submitted cases met the case definition. For this cohort study, we conducted a retrospective analysis of cases reported from 55 public health jurisdictions to the CDC national surveillance program as of April 4, 2023, with symptom onset on or before December 31, 2022, meeting the May 2020 case definition.^7,8^ We excluded cases with onset on January 1, 2023, or later that were adjudicated with the 2023 CDC and CSTE surveillance case definition.^9^ Data from free-text responses from the surveillance case report form were reviewed by clinicians and categorized appropriately. Race and ethnicity data were obtained from medical records at hospitalization, and patients were categorized as Hispanic or Latino, non-Hispanic Asian, non-Hispanic Black, non-Hispanic White, or other or multiple race (including American Indian, Alaskan Native or Aboriginal Canadian, Native Hawaiian, and Other Pacific Islander). These data were collected because of the association between MIS-C incidence and race and ethnicity early in the pandemic.^3,4^ This activity was reviewed by the CDC, deemed public health surveillance and not research, and was thus exempt from informed consent. This investigation was conducted in accordance with applicable federal law and CDC policy (eg, 45 CFR part 46.102(l)(2); 21 CFR part 56; 42 USC §241(d); 5 USC §552a; 44 USC §3501 et seq.). This report followed the Strengthening the Reporting of Observational Studies in Epidemiology (STROBE) reporting guideline.^26^

We described patient characteristics by SARS-CoV-2 variant predominance periods. Similar to prior analyses,^27^ periods were defined using 50% or more of SARS-CoV-2 variant proportions from US CDC national genomic surveillance,^28,29,30^ with an additional 2-week lag due to presumed minimal delay from SARS-CoV-2 infection to MIS-C onset: pre-Delta period (July 9, 2021, and earlier), Delta period (July 10 to December 31, 2021); and Omicron period (January 1, 2022, and later).

### Statistical Analysis

#### Indicator Variable Selection

We selected clinical signs and symptoms, diagnoses, complications, imaging results, and laboratory testing variables from the case report form for use as binary indicator (ie, input) variables in LCA (eAppendix in Supplement 1). To restrict to the most informative indicators, we removed variables with a high percentage (≥20%) of missing data, high correlation with other variables (Pearson correlation ≥0.5), and rare (≤10%) or high (≥90%) prevalence. Prevalence was calculated based on nonmissing data except for variables derived from echocardiographic results, where prevalence was calculated among all case patients. Missingness for echocardiographic results was likely not at random because selection of which patients underwent echocardiography could depend on clinical judgment. We created composite variables for overlapping fields, resulting in 29 final variables (eMethods in Supplement 1).

Cases with 10 or more missing variables (n = 389) were excluded, and multiple imputation of the 29 variables was conducted on all remaining 8944 cases (95.8% of all cases). Clinical outcomes of interest (ICU admission, ICU and hospitalization length of stay, and mortality), patient demographics, and comorbidities were not used as indicator variables or adjustment covariates and did not influence cluster assignment.

#### LCA and Data Interpretation

LCA was run using the poLCA R package (number of times to estimate the model set to 20 and maximum iterations set to 5000, with default parameters otherwise), and LCA combined with variable selection was run using the LCAvarsel package.^31,32^ The number of clusters was selected after considering multiple factors, including (1) model fit based on the bayesian information criterion and Akaike information criterion; (2) cluster distinctiveness using entropy and principal component analysis, an approach for identifying variables contributing the most variance^33^; (3) comparability with results from applying LCA with variable selection; (4) consistency of clustering solutions across dataset subsamples; and (5) clinical interpretability (eMethods in Supplement 1).

#### Cluster Interpretation

We interpreted clusters based on distinct clinical signs, symptoms, complications, and laboratory testing results, and we described differences between clusters by demographics, comorbidities, and clinical outcomes (ICU admission, ICU and hospitalization length of stay, and mortality). Two-sided *P* ≤ .05 from the χ^2^ test for categorical variables and Kruskal-Wallis test for continuous variables were interpreted as evidence that distributions were significantly different between clusters. Changes in cluster prevalence were described across SARS-CoV-2 predominant variant periods. To estimate differences in case ascertainment between the 2023 CSTE and CDC surveillance case definition and the 2020 case definition, we calculated the percentage of MIS-C cases in each cluster that would also meet the 2023 CSTE and CDC surveillance case definition, excluding cases without a reported quantitative C-reactive protein measurement. R software, version 4.1.3 (R Foundation) was used to conduct all analyses.

## Results

We analyzed 8944 MIS-C cases that met the inclusion criteria and were reported to the CDC using the 2020 CDC MIS-C case definition. Symptom onset ranged from February 19, 2020, through December 31, 2022. Case patients had a median (IQR) age of 8.7 (5.0-12.5) years; of 8940 patients with known sex, 5407 (60.5%) were male and 3533 (39.5%) were female. Of 8496 patients with known race and ethnicity information, 2202 (25.9%) were Hispanic or Latino, 248 (2.9%) were non-Hispanic Asian, 2576 (30.3%) were non-Hispanic Black, 3083 (36.3%) were non-Hispanic White, and 387 (4.6%) were other race or ethnicity or multiple races (Table). Preexisting conditions were reported for 2210 patients (24.7%), with the most common conditions being obesity (1287 of 7603 children aged >2 years [16.9%]), chronic lung disease (577 [6.5%]), and congenital malformations excluding congenital heart disease (362 [4.0%]). A total of 4727 patients with MIS-C (52.9%) were admitted to the ICU, and 70 (0.8%) died. Among included cases, 5188 (58.0%) were reported during the pre-Delta period, 2318 (25.9%) during the Delta period, and 1438 (16.1%) during the Omicron period. Among case patients in each variant period, 2979 (57.4%) were admitted to the ICU in the pre-Delta period, 1165 (50.3%) in the Delta period, and 583 (40.5%) in the Omicron period. The median (IQR) age decreased from 9.0 (5.2-13.0) years during the pre-Delta period to 7.2 (3.9-11.2) years during the Omicron period (*P* < .001).

**Table.** Characteristics of Patients With Multisystem Inflammatory Syndrome in Children by Predominant Circulating SARS-CoV-2 Variant, US, February 2020 to December 2022^a^

Characteristic	No. (%) of patients^b^			
	Pre-Delta (n = 5188)	Delta (n = 2318)	Omicron (n = 1438)	Overall (N = 8944)
Age, median (IQR), y^c^	9.0 (5.2-13.0)	9.1 (5.5-12.3)	7.2 (3.9-11.2)	8.7 (5.0-12.5)
Age category, y				
<1	141 (2.9)	64 (2.9)	56 (4.2)	261 (3.1)
1-4	1020 (21.3)	403 (18.6)	414 (31.4)	1837 (22.2)
5-9	1596 (33.3)	783 (36.0)	430 (32.6)	2809 (33.9)
10-14	1324 (27.6)	640 (29.5)	310 (23.5)	2274 (27.4)
15-20	715 (14.9)	282 (13.0)	109 (8.3)	1106 (13.3)
Sex^d^				
Female	2078 (40.1)	889 (38.4)	566 (39.4)	3533 (39.5)
Male	3110 (59.9)	1427 (61.6)	870 (60.6)	5407 (60.5)
Race and ethnicity^e^				
Hispanic or Latino	1542 (30.9)	395 (18.3)	265 (19.7)	2202 (25.9)
Non-Hispanic Asian	149 (3.0)	38 (1.8)	61 (4.5)	248 (2.9)
Non-Hispanic Black	1523 (30.5)	675 (31.2)	378 (28.1)	2576 (30.3)
Non-Hispanic White	1556 (31.2)	941 (43.5)	586 (43.5)	3083 (36.3)
Other or multiple race^f^	217 (4.4)	113 (5.2)	57 (4.2)	387 (4.6)
Any preexisting conditions	1389 (26.8)	553 (23.9)	268 (18.6)	2210 (24.7)
Obesity^g^	836 (18.9)	322 (15.9)	129 (11.1)	1287 (16.9)
Chronic lung disease	357 (6.9)	144 (6.2)	76 (5.3)	577 (6.5)
Other congenital malformations^h^	212 (4.1)	97 (4.2)	53 (3.7)	362 (4.0)
Seizures	105 (2.0)	39 (1.7)	32 (2.2)	176 (2.0)
Congenital heart disease	96 (1.9)	34 (1.5)	21 (1.5)	151 (1.7)
Immunosuppression	48 (0.9)	13 (0.6)	15 (1.0)	76 (0.8)
Type 1 or 2 diabetes	44 (0.8)	14 (0.6)	6 (0.4)	64 (0.7)
Sickle cell disease	26 (0.5)	20 (0.9)	6 (0.4)	52 (0.6)
ICU admission	2979 (57.4)	1165 (50.3)	583 (40.5)	4727 (52.9)
ICU length of stay, median (IQR), d^i^	4.0 (2.0-6.0)	3.0 (2.0-5.0)	3.0 (2.0-5.0)	3.0 (2.0-5.0)
Hospital length of stay, median (IQR), d^j^	6.0 (4.0-8.0)	5.0 (4.0-7.0)	5.0 (3.0-6.8)	5.0 (4.0-8.0)
Death	36 (0.7)	24 (1.0)	10 (0.7)	70 (0.8)

Abbreviation: ICU, intensive care unit.

^a^ Periods were defined using 50% or more of SARS-CoV-2 variant proportions from national genomic surveillance with an additional 2-week lag due to presumed minimal delay from SARS-CoV-2 infection to multisystem inflammatory syndrome in children onset: pre-Delta (July 9, 2021, and earlier); Delta (July 10 to December 31, 2021); and Omicron (January 1, 2022, and later).

^b^ Unless otherwise indicated.

^c^ A total of 657 patients were missing information on age.

^d^ A total of 4 patients were missing information on sex.

^e^ A total of 448 patients were missing information on race and ethnicity.

^f^ Other races include patients identifying as American Indian, Alaskan Native or Aboriginal Canadian, Native Hawaiian, and Other Pacific Islander.

^g^ Obesity assessed by clinician diagnosis of obesity or body mass index–based obesity; calculated only in 7603 children older than 2 years.

^h^ Including conditions such as fetal alcohol syndrome, Ehlers-Danlos syndrome, achondroplasia, and chromosomal abnormalities causing malformations.

^i^ Among 4727 patients admitted to the ICU, 1174 were missing information on ICU length of stay.

^j^ A total of 760 patients were missing information on hospitalization length of stay.

We conducted LCA on the 8944 cases using 29 clinical variables and identified 3 clusters as a suitable fit after considering multiple factors, including information criteria, cluster distinctiveness, and clinical interpretability (eFigures 1 and 2 and eTable 1 in Supplement 1). There were 713 cases (8.0%) in cluster 1, 3359 (37.6%) in cluster 2, and 4872 (54.5%) in cluster 3. We conducted sensitivity analyses on cluster robustness, finding that identified clusters were consistent when subsampling the dataset (eFigure 3 in Supplement 1) and similar to clusters obtained from applying LCA with variable selection (eMethods in Supplement 1).

**Figure 1.**
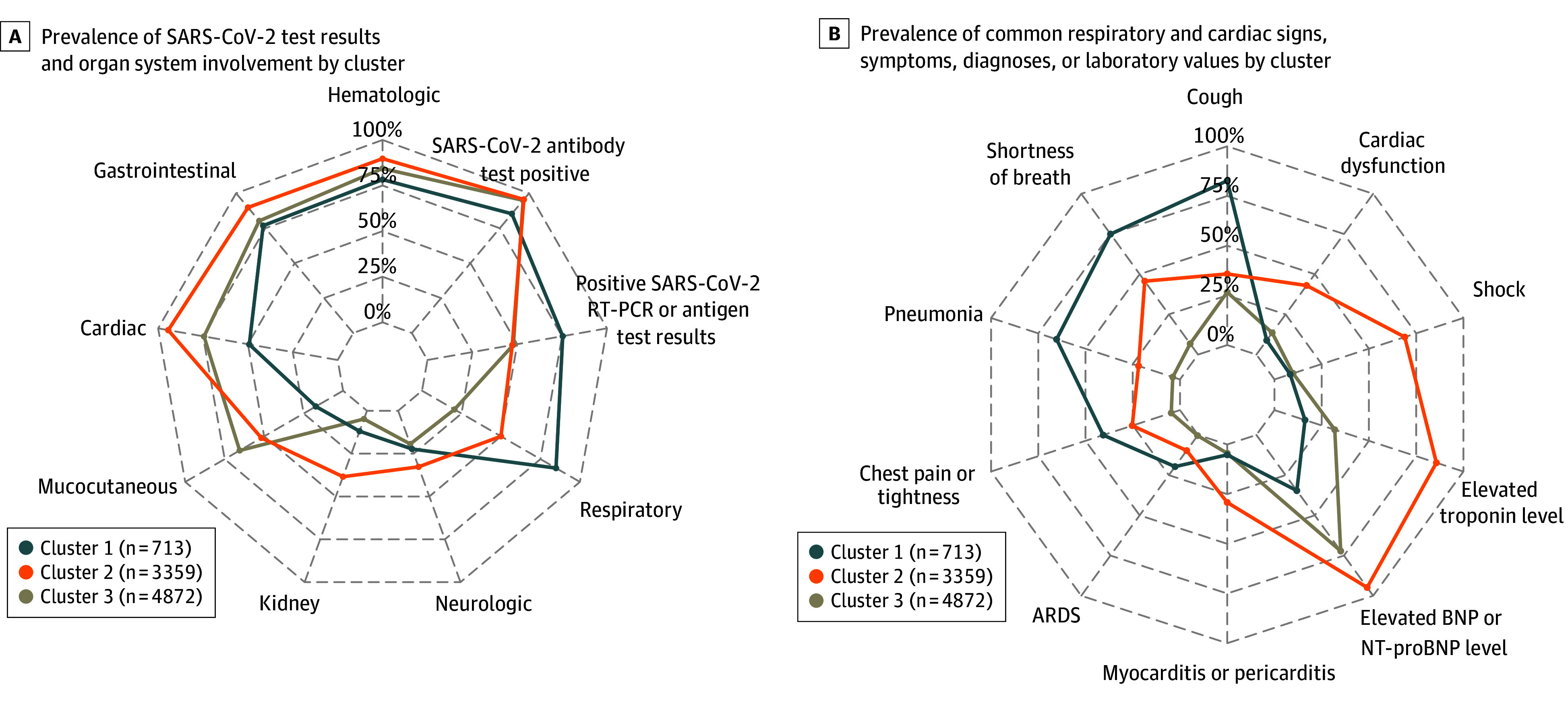
Prevalence of SARS-CoV-2 Test Results, Organ System Involvement, and Common Respiratory and Cardiac Presentations by Latent Class Analysis–Inferred Clusters Prevalence was calculated based on nonmissing data except for cardiac dysfunction, for which prevalence was calculated based on all patients in a cluster, because missingness of echocardiographic results was likely not at random. Sample sizes in cluster labels indicate total numbers of patients within each cluster. Cardiac dysfunction was assessed by left or right ventricular dysfunction on echocardiogram, and shock was indicated in a clinical note or receipt of vasopressors. Cluster 1 indicates the respiratory cluster; cluster 2, shock and cardiac cluster; and cluster 3, undifferentiated cluster. ARDS indicates acute respiratory distress syndrome; BNP, B-type natriuretic peptide; NT-proBNP, N-terminal prohormone of brain natriuretic peptide; RT-PCR, reverse transciptase–polymerase chain reaction.

Case patients in cluster 1, termed the respiratory cluster, were characterized by pronounced respiratory organ system involvement (Figure 1). Patients in this cluster had the highest prevalence of cough, shortness of breath, pneumonia, chest pain or tightness, and acute respiratory distress syndrome and a lower prevalence of mucocutaneous symptoms (Figure 1; eTable 2 in Supplement 1). Patients in this cluster also tended to be older (median [IQR] age, 12.7 [6.3-16.5] years), and the percentage of patients with 1 or more comorbidity was 41.1% (293 of 713) compared with 31.2% (1049 of 3359) in cluster 2 and 17.8% (868 of 4872) in cluster 3 (*P* < .001). Common comorbidities in cluster 1 patients included obesity (181 of 570 children aged >2 years [31.8%]), noncardiac congenital malformations (69 [9.7%]), and chronic lung disease (67 [9.4%]). The percentage of positive SARS-CoV-2 antigen or reverse transcriptase–polymerase chain reaction test results and preceding COVID-19–like illness were highest in cluster 1 (eTable 2 in Supplement 1).

**Figure 2.**
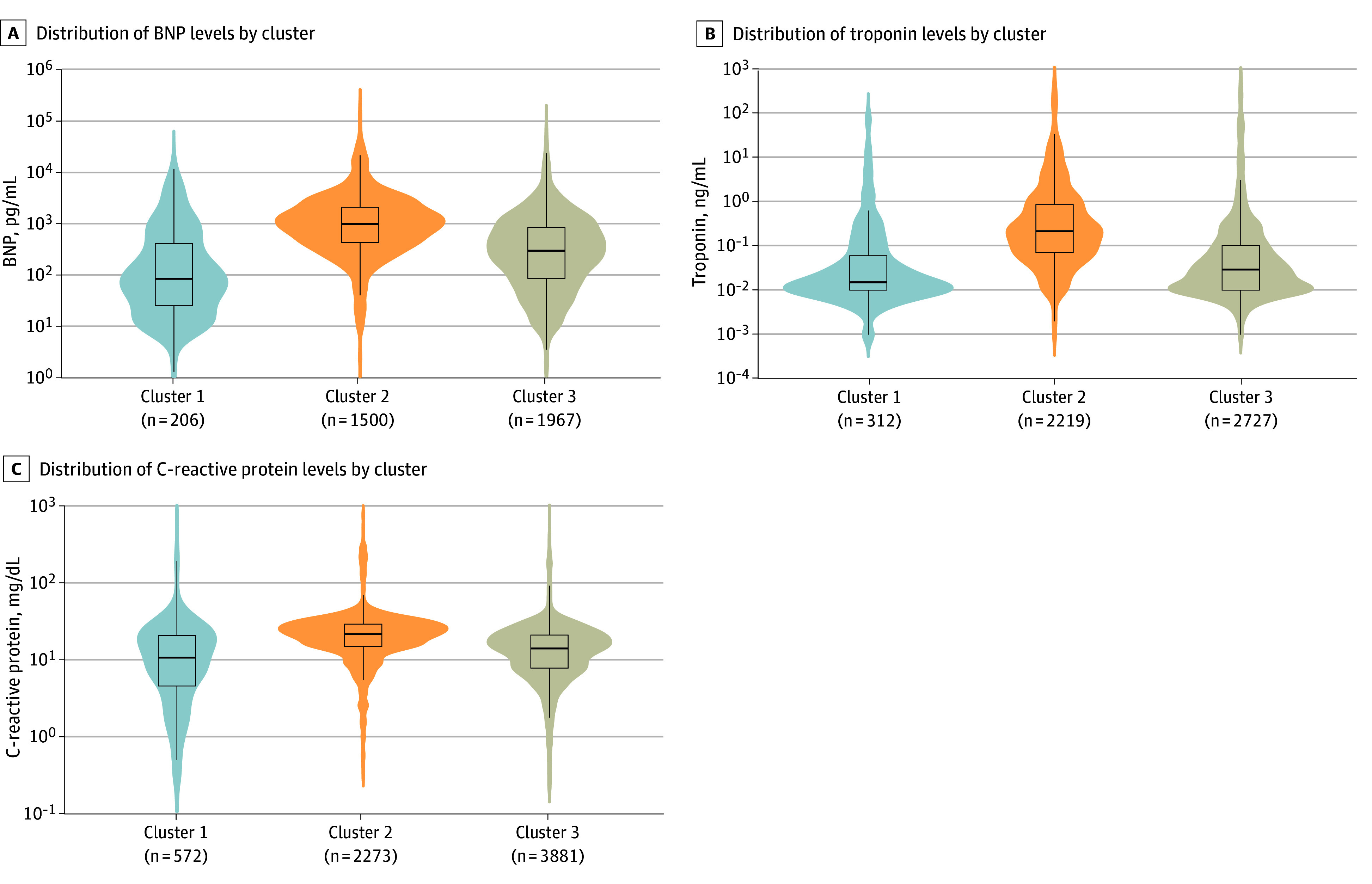
Distributions of Levels of B-Type Natriuretic Peptide (BNP), Troponin, and C-Reactive Protein by Latent Class Analysis–Inferred Clusters Distributions are scaled to have the same area. Horizontal lines indicate medians; edges of boxes, IQRs; and whiskers, upper and lower adjacent values. Sample sizes indicate the numbers of patients within each cluster with nonmissing data. Global differences across clusters were statistically significant (*P* < .001) as assessed using the Kruskal-Wallis test for continuous variables for all 3 clinical biomarkers. Cluster 1 indicates the respiratory cluster; cluster 2, shock and cardiac cluster; and cluster 3, undifferentiated cluster. To convert BNP to nanograms per liter, multiply by 1; to convert troponin to micrograms per liter, multiply by 1; to convert C-reactive protein to milligrams per liter, multiply by 10.

We designated cluster 2 as the shock and cardiac cluster because patients in this cluster had the highest prevalence of shock (as indicated in a clinical note or receipt of vasopressors; 69.0%) and reported cardiac involvement (94.4%), comprising shock, elevated troponin level, elevated B-type natriuretic peptide (BNP) level, abnormal echocardiographic results, or arrhythmia (Figure 1; eTable 2 in Supplement 1). Cluster 2 patients also had the highest prevalence of other cardiac complications, including myocarditis or pericarditis and cardiac dysfunction on echocardiogram. Among patients with available data, distributions of BNP (median [IQR], 966.4 [419.8-2050.0] pg/mL [to convert to nanograms per liter, multiply by 1]), troponin (median [IQR], 0.21 [0.07-0.87] ng/mL [to convert to micrograms per liter, multiply by 1]), and C-reactive protein (median [IQR], 21.0 [14.4-28.3] mg/dL [to convert to milligrams per liter, multiply by 10]) were skewed upward relative to other clusters (*P* < .001 for all) (Figure 2). Patients in this cluster had the largest number of organ systems involved (median [IQR], 5 [4-5] systems); the highest prevalence of hematologic, gastrointestinal, kidney, and neurologic involvement (Figure 1); and slightly younger age (median [IQR] age, 10.8 [7.7-14.0] years) compared with respiratory cluster patients (median [IQR] age, 12.7 [6.3-16.5] years) (*P* < .001).

Cluster 3 comprised the remaining patients with MIS-C, who were younger (median [IQR] age, 6.8 [3.6-10.3] years) and had fewer comorbidities (4004 patients [82.2%] did not report any comorbidities) (eTable 2 in Supplement 1). Organ system involvement in this cluster overlapped with the first 2 clusters, and we designated this the undifferentiated cluster (Figure 1). Cluster 3 patients had slightly higher prevalence of mucocutaneous symptoms (3192 [65.5%]) compared with cluster 2 patients (Figure 1), and 166 of 4872 patients (3.4%) met the clinical criteria for Kawasaki disease (presence of fever, rash, lesions, cervical lymphadenopathy, and conjunctival injection) vs 0 in cluster 1 and 67 of 3359 patients (2.0%) in cluster 2 (*P* < .001).

**Figure 3.**
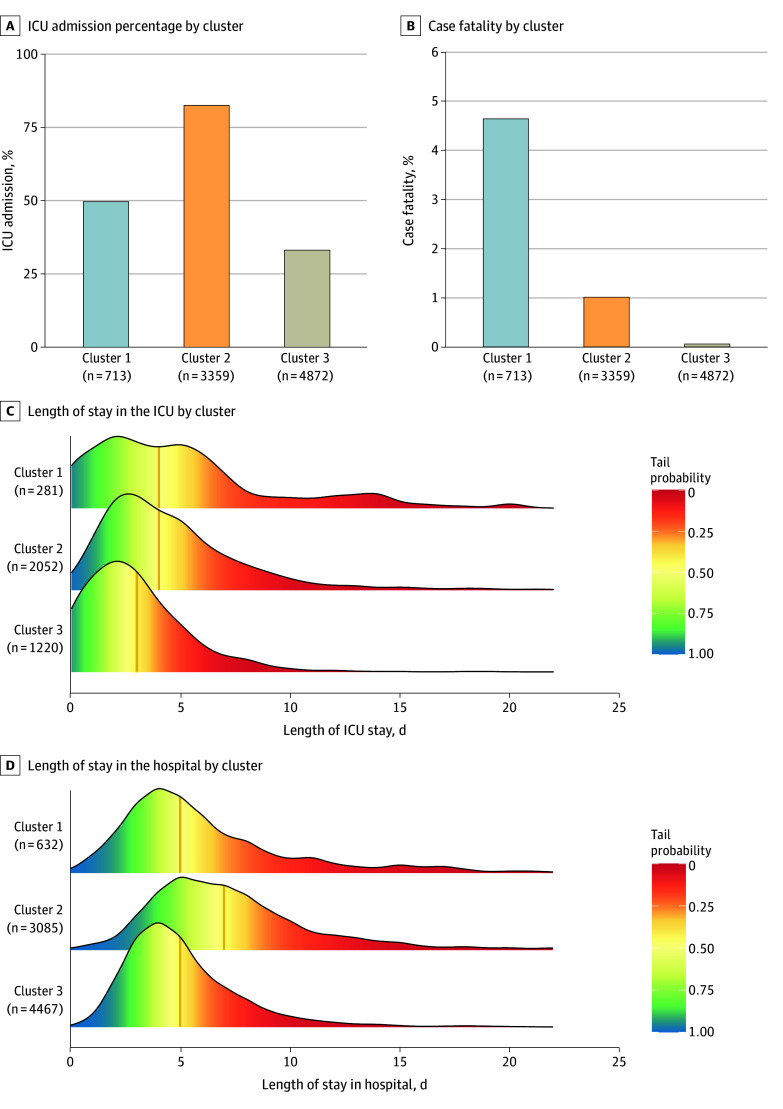
Intensive Care Unit (ICU) Admission, Case Fatality, and Length of Stay in the ICU and Hospital by Latent Class Analysis–Inferred Cluster Sample sizes indicate numbers of patients from each cluster included after removing patients with missing data. Tail probability is defined as the percentage of patients who were in the ICU (C) or hospitalized (D) beyond a certain length of time. Vertical orange line represents median length of stay (C and D).

Morbidity and mortality from MIS-C were concentrated in the respiratory cluster and shock and cardiac cluster. The percentage of patients with MIS-C admitted to the ICU was highest for the shock and cardiac cluster (82.3% [2765/3359]) followed by the respiratory (49.5% [353 of 713]) and undifferentiated clusters (33.0% [1609 of 4872]) (*P* < .001) (Figure 3). Similarly, among patients with data on length of stay available, 129 of 632 hospitalizations (20.4%) and 54 of 281 ICU stays (19.2%) in the respiratory cluster lasted 10 or more days compared with 708 of 3085 (22.9%) and 157 of 2052 (7.7%), respectively, in the shock and cardiac cluster and 293 of 4467 (6.6%) and 19 of 1220 (1.6%), respectively, in the undifferentiated cluster (Figure 3). The crude case fatality ratios were higher in the respiratory cluster (4.6%) and shock and cardiac cluster (1.0%) compared with the undifferentiated cluster (0.1%) (*P* < .001) (Figure 3), and most deaths (67 of 70 [95.7%]) occurred in these first 2 clusters.

**Figure 4.**
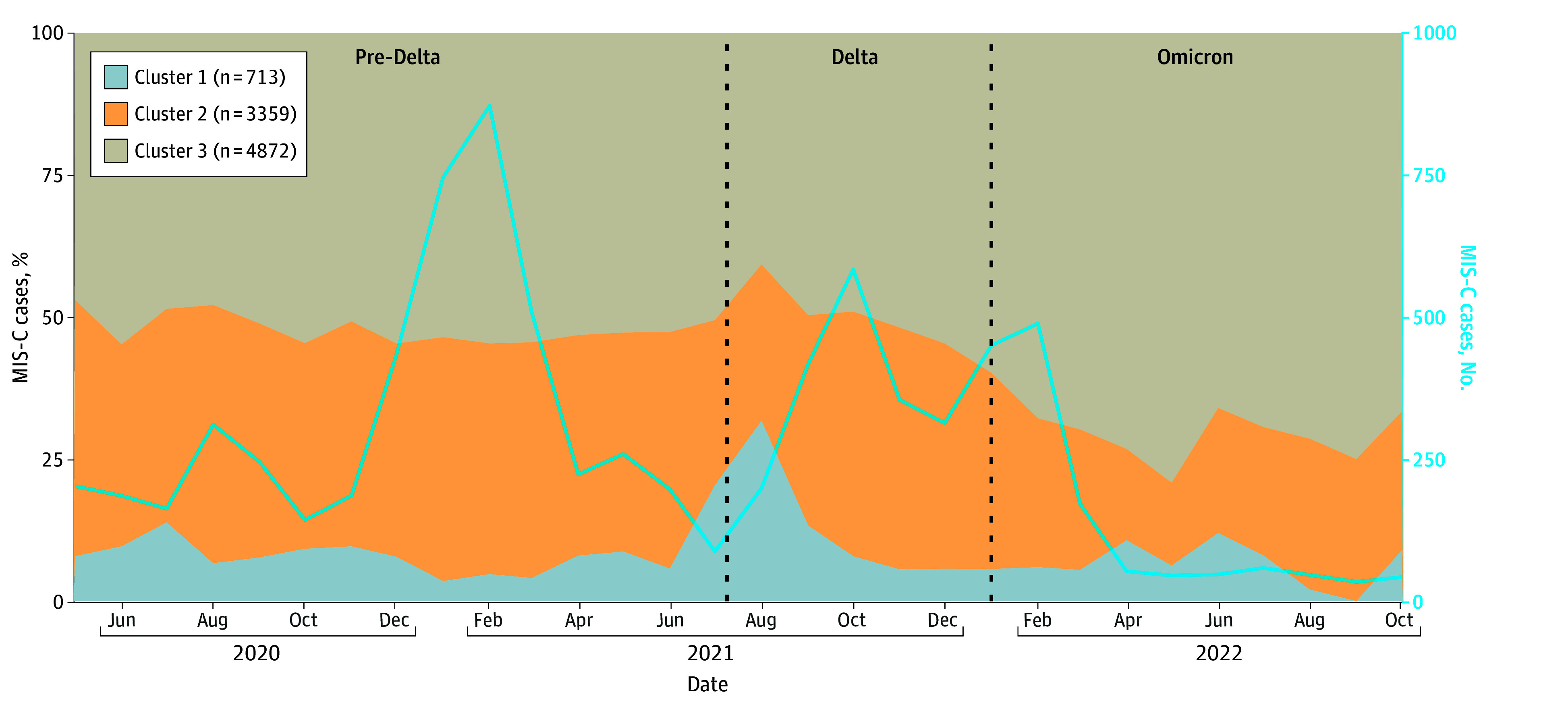
Monthly Percentages of Multisystem Inflammatory Syndrome in Children (MIS-C) Cases by Latent Class Analysis–Inferred Cluster and Number of Total MIS-C Cases, US, May 2020 to October 2022 Variant periods were defined using 50% or more of SARS-CoV-2 variant proportions from national genomic surveillance with an additional 2-week lag due to presumed minimal delay from SARS-CoV-2 infection to MIS-C onset: pre-Delta (July 9, 2021, and earlier), Delta (July 10 to December 31, 2021), and Omicron (January 1, 2022, and later).^28,29^ Months in which the total number of cases was less than 30 (April 2020 and earlier as well as November 2022 and later) were suppressed per National Center for Health Statistics standards.^30^

We characterized the changing epidemiology of MIS-C over time using LCA-derived clusters. The combined percentage of MIS-C cases in the respiratory cluster and shock and cardiac cluster was 53.0% (114 of 215) in May 2020; this percentage remained approximately stable (range, 45.2% [89 of 197 in June 2020] to 59.2% [125 of 211 in August 2021]) until the end of 2021. This combined percentage began to decrease substantially after emergence of the Omicron variant (Figure 4) and reached 33.3% (15 of 45) in October 2022; total counts of monthly MIS-C cases also decreased during this time.

To evaluate differences in case ascertainment between the 2020 CDC MIS-C case definition and the 2023 CSTE and CDC surveillance case definition, we calculated the percentage of MIS-C cases in each cluster that would meet the 2023 definition. The percentage of case patients who met the 2023 definition was highest for the shock and cardiac cluster (90.7% [2986 of 3293]) followed by the undifferentiated cluster (77.9% [3723 of 4779]); the percentage in the respiratory cluster was substantially lower (45.8% [325 of 709]) (*P* < .001). After restricting to only patients who met the 2023 case definition, the case fatality ratio among patients in the respiratory cluster (4.9%) and shock and cardiac cluster (1.0%) remained elevated compared with patients in the undifferentiated cluster (0%) (*P* < .001).

## Discussion

In this analysis, statistical clustering of patients with MIS-C from US national surveillance data with symptom onset from February 2020 to December 2022 identified 3 subgroups that differed in presentation, outcomes, temporal distribution, and alignment with the 2023 surveillance case definition. Patients in the respiratory cluster comprised older children with frequent comorbidities and a higher prevalence of COVID-19–like illness preceding MIS-C and positive antigen and reverse transcriptase–polymerase chain reaction test results. Patients in the shock and cardiac cluster tended to have the highest number of additional organ systems involved, whereas patients in the undifferentiated cluster had less defined organ system involvement and lower clinical severity.

The identification of clusters of MIS-C informs our understanding of heterogeneity in severity and changing epidemiology over time. Previous studies have identified individual risk factors for severity, including obesity, shortness of breath, abdominal pain, older age, and increased levels of cardiac dysfunction–associated biomarkers.^12,34^ Our findings support and synthesize these results by demonstrating that clinical severity and mortality are concentrated among 2 distinct classes of patients with MIS-C, defined by predominance of respiratory and shock or cardiac signs and symptoms, respectively. Patients in both clusters exhibited frequent preexisting conditions, including obesity. Additionally, decreases in the severity of MIS-C have been described over time,^18,19,35,36^ and we observed a concurrent decrease in respiratory cluster and shock and cardiac cluster prevalence after the emergence of the Omicron variant.

Our results also provide insight into categories of patients with MIS-C initially reported using the 2020 case definition that meet or do not meet criteria for the 2023 CSTE and CDC surveillance case definition.^7,9^ The respiratory organ involvement criterion was removed from the 2023 definition in an attempt to decrease misclassification of severe acute COVID-19 as MIS-C. Proportions of discordant patients between the 2020 and 2023 case definitions were highest for the respiratory cluster and lowest for the shock and cardiac cluster, indicating that the new case definition likely retains sensitivity and specificity for identifying severe MIS-C while potentially reducing misclassification from inclusion of severe acute pediatric COVID-19. Additionally, approximately half of respiratory cluster patients continued to meet the 2023 case definition, highlighting that MIS-C may have respiratory involvement or cardiac involvement that manifests as respiratory symptoms.

These findings align with and expand on previous statistical clustering analyses of MIS-C cases. Godfred-Cato et al^7^ identified 3 clusters among 570 early MIS-C cases, corresponding to typical MIS-C, MIS-C overlapping acute COVID-19, and MIS-C overlapping Kawasaki disease. Geva et al^10^ applied the partitioning around medoids algorithm on 1526 cases of acute COVID-19 and MIS-C, identifying a typical MIS-C cluster with high cardiovascular involvement and hyperinflammation, an acute COVID-19 cluster overlapping MIS-C, and a younger and less critically ill cluster. Rao et al^15^ analyzed 1139 children using LCA and identified classes corresponding to mild, moderate, and severe presentations. We identified an MIS-C cluster with frequent respiratory involvement similar to clusters identified by Godfred-Cato et al^7^ and Geva et al^10^ but with much lower relative frequency. We demonstrated that this relatively rare cluster was associated with increased clinical severity; because this analysis is the largest statistical clustering analysis of MIS-C cases to date, it may have been better powered to identify clusters with lower prevalence and characterize their association with mortality, a relatively rare outcome of MIS-C.

### Limitations

The findings in this report are subject to several limitations. First, because LCA is inherently an exploratory tool, these results are not intended to be used prognostically or for clinical decision-making. Second, the cluster labels we have used (eg, respiratory cluster and shock and cardiac cluster) are simplified descriptors; all clusters include some degree of respiratory or cardiac symptoms, and cases within clusters are not homogeneous. Third, we used the full range of clinical data available as input variables for LCA, but these correlated data present challenges for LCA, which we tried to address through multiple approaches (eMethods in Supplement 1). Fourth, national MIS-C case ascertainment relies on passive surveillance, and underreporting of MIS-C cases can occur. Fifth, these MIS-C cases were reported using the 2020 CDC MIS-C case definition, and clusters inferred here may not be fully generalizable to other countries with different case definitions or cases reported to the CDC in 2023 or later using the 2023 CDC and CSTE surveillance case definition.^2^

## Conclusion

MIS-C remains a public health concern that is likely to accompany surges in SARS-CoV-2 activity, as highlighted by relative increases in case counts during late 2023 compared with prior months.^23,24,37^ In this analysis, MIS-C cases reported to the CDC from 2020 to 2022 clustered into 3 groups with distinct symptoms and severity, highlighting the importance of recognizing the varied presentation of MIS-C. With additional validation, use of MIS-C phenotypic clusters in public health and clinical settings may be helpful in refining surveillance case definitions, contributing to our understanding of MIS-C pathophysiology, and assisting with recognizing the varied clinical presentations of MIS-C.

## Notes

Supplement 1. **eMethods.** Supplementary methods **eTable 1.** Model fit metrics (degrees of freedom, number of parameters, entropy, information metrics, log-likelihood) versus number of clusters **eTable 2.** Distribution of demographics, underlying health conditions, clinical signs, symptoms, complications and laboratory testing results, and clinical outcomes by latent class analysis-inferred clusters **eFigure 1.** Bayesian Information Criterion values (a) and entropy (b) versus number of clusters **eFigure 2.** Principal component analysis visualization of clustered MIS-C cases **eFigure 3.** Consistency of latent class analysis-inferred clusters estimated from sub-sampled datasets **eAppendix.** Multisystem inflammatory syndrome associated with COVID-19 case report form
eReferences

Supplement 2.
Data Sharing Statement

## References

[1] Patel JM. Multisystem inflammatory syndrome in children (MIS-C). Curr Allergy Asthma Rep. 2022;22(5):53-60. doi:10.1007/s11882-022-01031-4 35314921 PMC8938222

[2] Molloy EJ, Nakra N, Gale C, Dimitriades VR, Lakshminrusimha S. Multisystem inflammatory syndrome in children (MIS-C) and neonates (MIS-N) associated with COVID-19: optimizing definition and management. Pediatr Res. 2023;93(6):1499-1508. doi:10.1038/s41390-022-02263-w 36050390 PMC9436161

[3] Stierman B, Abrams JY, Godfred-Cato SE, . Racial and ethnic disparities in multisystem inflammatory syndrome in children in the United States, March 2020 to February 2021. Pediatr Infect Dis J. 2021;40(11):e400-e406. doi:10.1097/INF.0000000000003294 34382615 PMC8505134

[4] Payne AB, Gilani Z, Godfred-Cato S, ; MIS-C Incidence Authorship Group. Incidence of multisystem inflammatory syndrome in children among US persons infected with SARS-CoV-2. JAMA Netw Open. 2021;4(6):e2116420. doi:10.1001/jamanetworkopen.2021.16420 34110391 PMC8193431

[5] COVID data tracker MIS-C national surveillance. Centers for Disease Control and Prevention. Accessed March 13, 2024. https://covid.cdc.gov/covid-data-tracker/#mis-national-surveillance

[6] Feldstein LR, Tenforde MW, Friedman KG, ; Overcoming COVID-19 Investigators. Characteristics and outcomes of US children and adolescents with multisystem inflammatory syndrome in children (MIS-C) compared with severe acute COVID-19. JAMA. 2021;325(11):1074-1087. doi:10.1001/jama.2021.2091 33625505 PMC7905703

[7] Godfred-Cato S, Bryant B, Leung J, ; California MIS-C Response Team. COVID-19-associated multisystem inflammatory syndrome in children—United States, March-July 2020. MMWR Morb Mortal Wkly Rep. 2020;69(32):1074-1080. doi:10.15585/mmwr.mm6932e2 32790663 PMC7440126

[8] Multisystem inflammatory syndrome in children (MIS-C) associated with coronavirus disease 2019 (COVID-19). Centers for Disease Control and Prevention. September 21, 2021. Accessed March 13, 2024. https://web.archive.org/web/20230305233605/https://emergency.cdc.gov/han/2020/han00432.asp

[9] Melgar M, Lee EH, Miller AD, . Council of State and Territorial Epidemiologists/CDC surveillance case definition for multisystem inflammatory syndrome in children associated with SARS-CoV-2 infection—United States. MMWR Recomm Rep. 2022;71(4):1-14. doi:10.15585/mmwr.rr7104a1 36520808 PMC9762894

[10] Geva A, Patel MM, Newhams MM, ; Overcoming COVID-19 Investigators. Data-driven clustering identifies features distinguishing multisystem inflammatory syndrome from acute COVID-19 in children and adolescents. EClinicalMedicine. 2021;40:101112. doi:10.1016/j.eclinm.2021.101112 34485878 PMC8405351

[11] Godfred-Cato S, Abrams JY, Balachandran N, . Distinguishing multisystem inflammatory syndrome in children from COVID-19, Kawasaki disease and toxic shock syndrome. Pediatr Infect Dis J. 2022;41(4):315-323. doi:10.1097/INF.0000000000003449 35093995 PMC8919949

[12] Abrams JY, Oster ME, Godfred-Cato SE, . Factors linked to severe outcomes in multisystem inflammatory syndrome in children (MIS-C) in the USA: a retrospective surveillance study. Lancet Child Adolesc Health. 2021;5(5):323-331. doi:10.1016/S2352-4642(21)00050-X 33711293 PMC7943393

[13] Bowen A, Miller AD, Zambrano LD, . Demographic and clinical factors associated with death among persons <21 years old with multisystem inflammatory syndrome in children—United States, February 2020-March 2021. Open Forum Infect Dis. 2021;8(8):ofab388. doi:10.1093/ofid/ofab388 34409123 PMC8364981

[14] Calfee CS, Delucchi K, Parsons PE, Thompson BT, Ware LB, Matthay MA; NHLBI ARDS Network. Subphenotypes in acute respiratory distress syndrome: latent class analysis of data from two randomised controlled trials. Lancet Respir Med. 2014;2(8):611-620. doi:10.1016/S2213-2600(14)70097-9 24853585 PMC4154544

[15] Rao S, Jing N, Liu X, . Spectrum of severity of multisystem inflammatory syndrome in children: an EHR-based cohort study from the RECOVER program. Sci Rep. 2023;13(1):21005. doi:10.1038/s41598-023-47655-y 38017007 PMC10684592

[16] Day-Lewis M, Berbert L, Baker A, Dionne A, Newburger JW, Son MBF. Updated case definition of MIS-C and implications for clinical care. Pediatrics. 2024;153(2):e2023063259. doi:10.1542/peds.2023-063259 38204335

[17] Miller AD, Zambrano LD, Yousaf AR, ; MIS-C Surveillance Authorship Group. Multisystem inflammatory syndrome in children—United States, February 2020-July 2021. Clin Infect Dis. 2022;75(1):e1165-e1175. doi:10.1093/cid/ciab1007 34864955 PMC8689703

[18] Miller AD, Yousaf AR, Bornstein E, . Multisystem inflammatory syndrome in children during severe acute respiratory syndrome coronavirus 2 (SARS-CoV-2) Delta and Omicron variant circulation—United States, July 2021-January 2022. Clin Infect Dis. 2022;75(suppl 2):S303-S307. doi:10.1093/cid/ciac471 35684958 PMC9214171

[19] Cohen JM, Carter MJ, Ronny Cheung C, Ladhani S; Evelina PIMS-TS Study Group. Lower risk of multisystem inflammatory syndrome in children (MIS-C) with the Delta and Omicron variants of SARS-CoV-2. Clin Infect Dis. 2023;76(3):e518-e521. doi:10.1093/cid/ciac553 35788276 PMC9278259

[20] Sperotto F, Gutiérrez-Sacristán A, Makwana S, ; Consortium for Clinical Characterization of COVID-19 by EHR (4CE). Clinical phenotypes and outcomes in children with multisystem inflammatory syndrome across SARS-CoV-2 variant eras: a multinational study from the 4CE consortium. EClinicalMedicine. 2023;64:102212. doi:10.1016/j.eclinm.2023.102212 37745025 PMC10511777

[21] Lopez L, Burgner D, Glover C, ; Australian Vasculitis Working Group and Paediatric Active Enhanced Disease Surveillance (PAEDS) Network. Lower risk of multi-system inflammatory syndrome in children (MIS-C) with the Omicron variant. Lancet Reg Health West Pac. 2022;27:100604. doi:10.1016/j.lanwpc.2022.100604 36237982 PMC9541565

[22] Laird-Gion J, Dionne A, Gauvreau K, . MIS-C across three SARS-CoV-2 variants: changes in COVID-19 testing and clinical characteristics in a cohort of U.S. children. Eur J Pediatr. 2023;182(6):2865-2872. doi:10.1007/s00431-023-04968-4 37055630 PMC10101535

[23] Hensley M, Jaggi P, Oster ME. Not gone, and should not be forgotten: multisystem inflammatory syndrome in children. Pediatr Infect Dis J. 2024;43(6):e218-e220. doi:10.1097/INF.0000000000004272 38295224

[24] Yousaf AR, Lindsey KN, Wu MJ, ; MIS-C Surveillance Authorship Group. Notes from the field: surveillance for multisystem inflammatory syndrome in children—United States, 2023. MMWR Morb Mortal Wkly Rep. 2024;73(10):225-228. doi:10.15585/mmwr.mm7310a2 38488279 PMC10948191

[25] Information for healthcare providers about multisystem inflammatory syndrome in children (MIS-C). Centers for Disease Control and Prevention. October 4, 2023. Accessed January 25, 2024. https://www.cdc.gov/mis/hcp/case-definition-reporting/index.html

[26] von Elm E, Altman DG, Egger M, Pocock SJ, Gøtzsche PC, Vandenbroucke JP; STROBE Initiative. Strengthening the Reporting of Observational Studies in Epidemiology (STROBE) statement: guidelines for reporting observational studies. BMJ. 2007;335(7624):806-808. doi:10.1136/bmj.39335.541782.AD 17947786 PMC2034723

[27] Zambrano LD, Newhams MM, Olson SM, ; Overcoming COVID-19 Investigators. BNT162b2 mRNA vaccination against coronavirus disease 2019 is associated with a decreased likelihood of multisystem inflammatory syndrome in children aged 5-18 years-United States, July 2021-April 2022. Clin Infect Dis. 2023;76(3):e90-e100. doi:10.1093/cid/ciac637 35924406 PMC9384630

[28] Lambrou AS, Shirk P, Steele MK, ; Strain Surveillance and Emerging Variants Bioinformatic Working Group; Strain Surveillance and Emerging Variants NS3 Working Group. Genomic surveillance for SARS-CoV-2 variants: predominance of the Delta (B.1.617.2) and Omicron (B.1.1.529) variants—United States, June 2021-January 2022. MMWR Morb Mortal Wkly Rep. 2022;71(6):206-211. doi:10.15585/mmwr.mm7106a4 35143464 PMC8830620

[29] Ma KC, Shirk P, Lambrou AS, . Genomic surveillance for SARS-CoV-2 variants: circulation of Omicron lineages—United States, January 2022-May 2023. MMWR Morb Mortal Wkly Rep. 2023;72(24):651-656. doi:10.15585/mmwr.mm7224a2 37319011 PMC10328465

[30] Parker JD, Talih M, Malec DJ. *National Center for Health Statistics Data Presentation Standards for Proportions.* National Center for Health Statistics, Centers for Disease Control and Prevention, US Department of Health and Human Services; 2017. 30248016

[31] Fop M, Smart KM, Murphy TB. Variable selection for latent class analysis with application to low back pain diagnosis. Ann Appl Stat. 2017;11(4):2080-2110. doi:10.1214/17-AOAS1061

[32] Linzer DA, Lewis JB. poLCA: an R package for polytomous variable latent class analysis. J Stat Softw. 2011;42:1-29. doi:10.18637/jss.v042.i10

[33] Greenacre M, Groenen PJF, Hastie T, D’Enza AI, Markos A, Tuzhilina E. Principal component analysis. Nat Rev Methods Primers. 2022;2(1):1-21. doi:10.1038/s43586-022-00184-w

[34] Khoury M, Harahsheh AS, Raghuveer G, ; International Kawasaki Disease Registry. Obesity and outcomes of Kawasaki disease and COVID-19–related multisystem inflammatory syndrome in children. JAMA Netw Open. 2023;6(12):e2346829. doi:10.1001/jamanetworkopen.2023.46829 38064213 PMC10709775

[35] Molloy MJ, Auger KA, Hall M, . Epidemiology and severity of illness of MIS-C and Kawasaki disease during the COVID-19 pandemic. Pediatrics. 2023;152(5):e2023062101. doi:10.1542/peds.2023-062101 37791428 PMC10598633

[36] Levy N, Koppel JH, Kaplan O, . Severity and incidence of multisystem inflammatory syndrome in children during 3 SARS-CoV-2 pandemic waves in Israel. JAMA. 2022;327(24):2452-2454. doi:10.1001/jama.2022.8025 35588048 PMC9121298

[37] Urgent need to increase immunization coverage for influenza, COVID-19, and RSV and use of authorized/approved therapeutics in the setting of increased respiratory disease activity during the 2023–2024 winter season. Centers for Disease Control and Prevention. December 14, 2023. Accessed January 25, 2024. https://emergency.cdc.gov/han/2023/han00503.asp

