# Supplementary material for: Phenotypic Classification of Multisystem Inflammatory Syndrome in Children Using Latent Class Analysis
Source: JAMA Netw Open. 2025 Jan 28;8(1):e2456272. doi: 10.1001/jamanetworkopen.2024.56272 (PMC11775748; doi:10.1001/jamanetworkopen.2024.56272)
Supplement: Supplement 1. — eMethods. Supplementary methods eTable 1. Model fit metrics (degrees of freedom, number of parameters, entropy, information metrics, log-likelihood) versus number of clusters eTable 2. Distribution of demographics, underlying health conditions, clinical signs, symptoms, complications and laboratory testing results, and clinical outcomes by latent class analysis-inferred clusters eFigure 1. Bayesian Information Criterion values (a) and entropy (b) versus number of clusters eFigure 2. Principal component analysis visualization of clustered MIS-C cases eFigure 3. Consistency of latent class analysis-inferred clusters estimated from sub-sampled datasets eAppendix. Multisystem inflammatory syndrome associated with COVID-19 case report form eReferences [file jamanetwopen-e2456272-s001.pdf]

Supplementary Online Content

Ma KC, Yousaf AR, Miller A, et al. Phenotypic classification of multisystem inflammatory syndrome in children using latent class analysis. *JAMA Netw Open*. 2024;8(1):e2456272. doi:10.1001/jamanetworkopen.2024.56272

eMethods. Supplementary methods
eTable 1. Model fit metrics (degrees of freedom, number of parameters, entropy, information metrics, log-likelihood) versus number of clusters
eTable 2. Distribution of demographics, underlying health conditions, clinical signs, symptoms, complications and laboratory testing results, and clinical outcomes by latent class analysis-inferred clusters
eFigure 1. Bayesian Information Criterion values (a) and entropy (b) versus number of clusters
eFigure 2. Principal component analysis visualization of clustered MIS-C cases
eFigure 3. Consistency of latent class analysis-inferred clusters estimated from sub-sampled datasets
eAppendix. Multisystem inflammatory syndrome associated with COVID-19 case report form
eReferences

This supplementary material has been provided by the authors to give readers additional information about their work.

© 2025 Ma KC et al. *JAMA Network Open*.

eMethods. Supplementary Methods

Composite variables

We created composite variables combining identical symptoms present in more than one section of the 2020 MIS-C case report form (Supplementary File 1). The clinical sign or symptom was considered present if present in any of the aggregated variables:

- Shock: shock during present illness (3.4.1.1), shock as a complication (4.11)
- Pneumonia: pneumonia during present illness (3.4.3.4), pneumonia as a complication (4.8), pneumonia from chest imaging (6.7.2)
- Acute respiratory distress syndrome (ARDS): ARDS during present illness (3.4.3.5), ARDS as a complication (4.7)
- Meningitis or encephalitis: meningitis during present illness (3.4.7.5), encephalitis or aseptic meningitis as a complication (4.10)
- SARS-CoV-2 PCR testing results: inclusion criteria (1.6.1), SARS-CoV-2 testing section (6.8)
- SARS-CoV-2 antibody testing results: inclusion criteria (1.6.2), SARS-CoV-2 testing section (6.10, 6.11, 6.12)
- SARS-CoV-2 antigen testing results: inclusion criteria (1.6.3), SARS-CoV-2 testing section (6.9)

Indicator variables and excluded variables list

We removed variables with a high percentage (≥20%) of missing data, high correlation with other indicator variables (as defined by Pearson correlation ≥0.5), and rare (≤10%) or high (≥90%) prevalence. The final list of binary variables used in the LCA model fitting process were:

- Shock
- Elevated troponin
- Elevated BNP or NT-proBNP
- Arrhythmia
- Myocarditis
- Acute kidney injury
- Cough
- Shortness of breath

© 2025 Ma KC et al. *JAMA Network Open*.

- Chest pain/tightness
- Pneumonia
- Acute respiratory distress syndrome
- Elevated D-dimer
- Thrombophilia
- Thrombocytopenia
- Abdominal pain
- Vomiting
- Diarrhea
- Elevated bilirubin
- Elevated liver enzymes
- Rash
- Mucocutaneous lesions
- Headache
- Altered mental state
- Syncope/near syncope
- Neck pain
- Myalgia
- Conjunctival injection
- Periorbital edema
- Cervical lymphadenopathy >1.5 cm diameter

The following variables were removed after meeting exclusion criteria:

- High missingness rates (≥20%): ventricular arrhythmia, supraventricular arrhythmia, other arrhythmia
- Low prevalence (≤10%): stroke, liver failure, pulmonary embolism, pericarditis, meningitis or encephalitis, encephalopathy, renal failure, congestive heart failure
- High prevalence (≥90%): fever
- High correlation: hypotension (highly correlated with shock)

© 2025 Ma KC et al. *JAMA Network Open*.

Cluster solution selection for latent class analysis

We opted to use the full range of clinical signs, symptoms, complications and laboratory testing results available from the MIS-C national surveillance case reporting form as input variables for LCA without collapsing data into broad organ system categories, as has been done in previous LCA analyses. Collapsing multiple symptoms into one category loses information on both severity (e.g., a patient reporting cough is treated equally as a patient experiencing acute respiratory distress syndrome) and the number of symptoms occurring within organ system groups. Additionally, organ system groupings are not distinct boundaries, and symptoms can overlap multiple organ systems. However, conducting LCA on all available symptom data presents challenges, including possible violation of the conditional independence assumption and the identification of spurious latent classes. We therefore used multiple approaches to select the number of clusters and assess robustness of our modeling approach:

1) Information criteria: identifying the number of clusters corresponding to a minimal information criterion is often used to identify best fit LCA models [1]. We observed that with the size of the dataset and number of indicator variables, the Bayesian Information Criterion (BIC) continued to decrease for each additional class added at least up until ten clusters. Instead of a local minimum, we instead looked for an inflection point in the BIC curve (Supplementary Figure 1) as has been suggested in the literature [1]; we observed that decreases in BIC began to diminish beginning with clusters of size three to five. Similar behavior was observed for other information criteria metrics, including the sample-size adjusted BIC, Akaike Information Criterion (AIC), and consistent AIC (CAIC) (Supplementary Table 1).
2) Cluster distinctiveness: we calculated relative entropy, which is a measure of cluster distinctiveness ranging from 0 to 1 with higher values indicating greater separation between clusters [1]. We found that a solution with three clusters had the highest entropy (Supplementary Table 1; Supplementary Figure 1). We also used principal component analysis, a variable reduction approach intended to simplify complex datasets into a smaller number of variables (i.e., principal components) that contribute the most variance [2]. PCA indicated visual separation in a plot of MIS-C cases along principal components one (x-axis) and two (y-axis) between a three-cluster solution inferred using LCA (Supplementary Figure 2). However, principal components one and two explain only ~17% of the total variance, and therefore are a limited representation of the full variation in MIS-C clinical phenotypes.

© 2025 Ma KC et al. *JAMA Network Open*.

3) Variable selection: we conducted LCA combined with variable selection to characterize the effect of reducing the number of indicator variables on model fit [3]. After running backwards selection with variable swapping via the *LCAvarsel* R package, 12 indicator variables remained. BIC indicated a solution with three or four clusters had the best fit and entropy was again highest for the three-cluster solution, supporting results from LCA model inference conducted without variable selection. However, overall entropy for all solutions was low (<0.55) indicating inferred clusters were not sufficiently separated, so we did not use these cluster assignments for final analyses.
4) Stability assessment: we evaluated how consistent clusters were when subsampling data, preferring solutions with cluster sizes that were less sensitive to changes in the input data, as our case patients come from passive surveillance and thus are likely a subsample of all MIS-C cases in the U.S. We randomly subsampled n=2000 cases from the full dataset 100 times and ran LCA varying the number of clusters from 2 to 6. For each cluster number, we used the subsampled LCA models to predict class membership for the full dataset and then computed the Adjusted Rand Index, a measure of similarity between two clustering assignments with 1 indicating identical results, for each pair of subsample-derived models. This allowed us to characterize the stability of inferred clusters. We observed that LCA solutions with cluster sizes of two and three produced the most consistent estimates, as measured by higher Rand Index values, when the input data were varied in this manner (Supplementary Figure 3).
5) Clinical interpretability: Based on clinical expertise of coauthors and colleagues, we determined that the solutions with two to four clusters generally corresponded to interpretable and clinically useful categories.

We selected the LCA solution with three clusters for further investigation on the basis of these criteria: diminishing returns in BIC, maximum entropy, results from LCA integrating variable selection, consistency under subsampling, and clinical interpretability.

© 2025 Ma KC et al. *JAMA Network Open*.

eTable 1. Model fit metrics (degrees of freedom, number of parameters, entropy, information metrics, log-likelihood) versus number of clusters.

Clusters	Degrees of freedom	Num params	entropy	BIC	ABIC	AIC	CAIC	Log-likelihood
1	8915	29		273092.7	273000.6	272886.9	273121.7	-136414.4
2	8885	59	0.6930307	265737.0	265549.5	265318.1	265796.0	-132600.1
3	8855	89	0.7673455	262591.2	262308.4	261959.4	262680.2	-130890.7
4	8825	119	0.6989315	261248.1	260869.9	260403.3	261367.1	-130082.7
5	8795	149	0.6846483	260239.2	259765.7	259181.5	260388.2	-129441.8
6	8765	179	0.6781580	259566.6	258997.8	258295.9	259745.6	-128969.0
7	8735	209	0.6713617	259120.8	258456.6	257637.1	259329.8	-128609.6
8	8705	239	0.6625687	258826.2	258066.7	257129.6	259065.2	-128325.8
9	8675	269	0.6671141	258652.4	257797.6	256742.8	258921.4	-128102.4
10	8645	299	0.6622905	258509.1	257558.9	256386.6	258808.1	-127894.3

Vuong-Lo-Mendell-Rubin test *P*-values were <0.0001 for all clusters assessed.

Abbreviations: BIC = Bayesian Information Criterion, ABIC = Sample-size Adjusted Bayesian Information Criterion, AIC = Akaike Information Criterion, CAIC = Consistent Akaike Information Criterion.

© 2025 Ma KC et al. *JAMA Network Open*.

eTable 2. Distribution of demographics, underlying health conditions, clinical signs, symptoms, complications and laboratory testing results, and clinical outcomes by latent class analysis-inferred clusters.

	1. Respiratory (N=713)	2. Shock/Cardiac (N=3359)	3. Undifferentiated (N=4872)	Chi-squared test statistic[a]	P-value[a]
**Age[b]**					<0.0001[c]
Median (IQR)	12.7 (6.3, 16.5)	10.8 (7.7, 14.0)	6.8 (3.6, 10.3)		
**Age group**				1400	<0.0001
<1 y	45 (7.0%)	16 (0.5%)	200 (4.5%)		
1-4 y	96 (14.8%)	283 (9.0%)	1458 (32.5%)		
5-9 y	99 (15.3%)	1083 (34.3%)	1627 (36.3%)		
10-14 y	162 (25.0%)	1207 (38.2%)	905 (20.2%)		
15-20 y	245 (37.9%)	569 (18.0%)	292 (6.5%)		
**Sex[d]**				7	0.03
Female	300 (42.1%)	1271 (37.9%)	1962 (40.3%)		
Male	413 (57.9%)	2086 (62.1%)	2908 (59.7%)		
**Race/ethnicity[e]**				137	<0.0001
Hispanic or Latino	209 (30.1%)	757 (23.7%)	1236 (26.8%)		
Non-Hispanic Asian	22 (3.2%)	83 (2.6%)	143 (3.1%)		
Non-Hispanic Black	201 (29.0%)	1194 (37.4%)	1181 (25.6%)		
Non-Hispanic White	241 (34.7%)	1019 (31.9%)	1823 (39.5%)		
Other/Multiple race	21 (3.0%)	139 (4.4%)	227 (4.9%)		
**Any pre-existing conditions**	293 (41.1%)	1049 (31.2%)	868 (17.8%)	304	<0.0001
**Obesity[f]**	181/570 (31.8%)	683/3109 (22.0%)	423/3924 (10.8%)	295	<0.0001
**Chronic lung disease**	67 (9.4%)	274 (8.2%)	236 (4.8%)	47	<0.0001
**Other congenital malformations[g]**	69 (9.7%)	122 (3.6%)	171 (3.5%)	63	<0.0001

© 2025 Ma KC et al. *JAMA Network Open*.

	1. Respiratory (N=713)	2. Shock/Cardiac (N=3359)	3. Undifferentiated (N=4872)	Chi-squared test statistic[a]	P-value[a]
**Seizures**	35 (4.9%)	65 (1.9%)	76 (1.6%)	36	<0.0001
**Congenital heart disease**	23 (3.2%)	76 (2.3%)	52 (1.1%)	28	<0.0001
**Immunosuppression**	23 (3.2%)	23 (0.7%)	30 (0.6%)	52	<0.0001
**Diabetes mellitus type 1 or 2**	11 (1.5%)	37 (1.1%)	16 (0.3%)	24	<0.0001
**Sickle cell disease**	14 (2.0%)	18 (0.5%)	20 (0.4%)	26	<0.0001
**ICU admission**	353 (49.5%)	2765 (82.3%)	1609 (33.0%)	1942	<0.0001
**Death**	33 (4.6%)	34 (1.0%)	3 (0.1%)	171	<0.0001
**ICU length of stay**					<0.0001[c]
Median (IQR)	4.00 (2.00, 7.00)	4.00 (2.00, 6.00)	3.00 (1.00, 4.00)		
**Hospital length of stay**					<0.0001[h]
Median (IQR)	5.00 (4.00, 8.00)	7.00 (5.00, 9.00)	5.00 (3.00, 6.00)		
**Cardiac involvement**	351 (49.2%)	3170 (94.4%)	3627 (74.4%)	947	<0.0001
**Renal involvement**	85 (11.9%)	1292 (38.5%)	234 (4.8%)	1545	<0.0001
**Respiratory involvement**	605 (84.9%)	1679 (50.0%)	1001 (20.5%)	1514	<0.0001
**Hematologic involvement**	558 (78.3%)	3014 (89.7%)	4109 (84.3%)	85	<0.0001
**Gastrointestinal involvement**	548 (76.9%)	3018 (89.8%)	3910 (80.3%)	159	<0.0001
**Mucocutaneous involvement**	123 (17.3%)	1738 (51.7%)	3192 (65.5%)	639	<0.0001
**Neurologic involvement**	159 (22.3%)	1099 (32.7%)	945 (19.4%)	192	<0.0001

© 2025 Ma KC et al. *JAMA Network Open*.

	1. Respiratory (N=713)	2. Shock/Cardiac (N=3359)	3. Undifferentiated (N=4872)	Chi-squared test statistic	P-value
**Number of organ systems involved**					<0.0001[h]
Median (IQR)	3.00 (3.00, 4.00)	5.00 (4.00, 5.00)	4.00 (3.00, 4.00)		
**Preceding COVID-19-like illness**	426/622 (68.5%)	1413/2835 (49.8%)	2052/4099 (50.1%)	78	<0.0001
**Positive SARS-CoV-2 RT-PCR or antigen test results**	520/690 (75.4%)	1514/3204 (47.3%)	2195/4587 (47.9%)	196	<0.0001
**SARS-CoV-2 antibody test positive**	378/443 (85.3%)	2999/3138 (95.6%)	4271/4499 (94.9%)	82	<0.0001
**Arrythmia**	148/702 (21.1%)	1055/3318 (31.8%)	788/4817 (16.4%)	269	<0.0001
**Myocarditis**	23/707 (3.3%)	936/3307 (28.3%)	173/4826 (3.6%)	1137	<0.0001
**Shock**	59/710 (8.3%)	2310/3349 (69.0%)	479/4865 (9.8%)	3389	<0.0001
**Elevated troponin**	109/674 (16.2%)	2796/3262 (85.7%)	1488/4662 (31.9%)	2580	<0.0001
**Elevated BNP or NT-proBNP**	215/621 (34.6%)	2694/2840 (94.9%)	2946/4080 (72.2%)	1217	<0.0001
**Acute kidney injury**	82/712 (11.5%)	1389/3334 (41.7%)	193/4823 (4.0%)	1861	<0.0001
**Cough**	582/704 (82.7%)	1192/3330 (35.8%)	1292/4853 (26.6%)	859	<0.0001
**Shortness of breath**	523/698 (74.9%)	1523/3337 (45.6%)	326/4841 (6.7%)	2426	<0.0001
**Chest pain/tightness**	280/689 (40.6%)	840/3326 (25.3%)	224/4833 (4.6%)	1026	<0.0001
**Pneumonia**	465/712 (65.3%)	734/3348 (21.9%)	186/4870 (3.8%)	1960	<0.0001
**ARDS**	140/711 (19.7%)	325/3347 (9.7%)	20/4870 (0.4%)	640	<0.0001
**Elevated d-dimer**	551/684 (80.6%)	3054/3240 (94.3%)	4099/4638 (88.4%)	146	<0.0001
**Thrombophilia**	90/686 (13.1%)	534/3193 (16.7%)	574/4609 (12.5%)	29	<0.0001
**Thrombocytopenia**	135/701 (19.3%)	1837/3304 (55.6%)	1622/4794 (33.8%)	530	<0.0001
**Abdominal pain**	290/688 (42.2%)	2750/3337 (82.4%)	2921/4826 (60.5%)	645	<0.0001
**Vomiting**	395/709 (55.7%)	2622/3346 (78.4%)	2955/4853 (60.9%)	318	<0.0001
**Diarrhea**	286/709 (40.3%)	2240/3344 (67.0%)	2297/4850 (47.4%)	366	<0.0001
**Elevated bilirubin**	102/699 (14.6%)	1175/3287 (35.7%)	641/4784 (13.4%)	593	<0.0001

© 2025 Ma KC et al. *JAMA Network Open*.

	1. Respiratory (N=713)	2. Shock/Cardiac (N=3359)	3. Undifferentiated (N=4872)	Chi-squared test statistic	P-value
**Elevated liver enzymes**	336/702 (47.9%)	2290/3303 (69.3%)	2046/4802 (42.6%)	569	<0.0001
**Rash**	116/713 (16.3%)	1722/3350 (51.4%)	3143/4853 (64.8%)	636	<0.0001
**Lesions**	14/709 (2.0%)	624/3329 (18.7%)	1278/4828 (26.5%)	245	<0.0001
**Headache**	222/690 (32.2%)	1925/3322 (57.9%)	1842/4829 (38.1%)	362	<0.0001
**Altered mental state**	69/704 (9.8%)	669/3328 (20.1%)	247/4840 (5.1%)	451	<0.0001
**Syncope/near syncope**	44/700 (6.3%)	297/3268 (9.1%)	103/4692 (2.2%)	190	<0.0001
**Neck pain**	28/681 (4.1%)	1082/3254 (33.3%)	1002/4699 (21.3%)	314	<0.0001
**Myalgia**	164/694 (23.6%)	1370/3317 (41.3%)	1103/4798 (23.0%)	328	<0.0001
**Conjunctival injection**	43/713 (6.0%)	1961/3346 (58.6%)	2874/4856 (59.2%)	742	<0.0001
**Periorbital edema**	9/700 (1.3%)	380/3262 (11.6%)	668/4672 (14.3%)	98	<0.0001
**Cervical lymphadenopathy**	22/655 (3.4%)	457/2848 (16.0%)	764/4097 (18.6%)	97	<0.0001

[a] Statistical significance of global differences across clusters was assessed using the Chi-squared test for categorical variables and Kruskal-Wallis test for continuous variables. Chi-squared test statistics were included for categorical variables.

[b] A total of 66 patients in cluster 1, 201 patients in cluster 2, and 390 patients in cluster 3 were missing information on age.

[c] Pairwise differences between all groups were statistically significant at the *P*=0.05 level using Dunn's test for multiple comparisons, except for the respiratory versus shock/cardiac cluster comparison.

[d] A total of 2 patients in cluster 2 and 2 patients in cluster 3 were missing information on sex.

[e] A total of 19 patients in cluster 1, 167 patients in cluster 2, and 262 patients in cluster 3 were missing information on race and ethnicity. Other races include patients identifying as American Indian, Alaskan Native or Aboriginal Canadian, Native Hawaiian, and other Pacific Islander.

© 2025 Ma KC et al. *JAMA Network Open*.

[f] Obesity assessed by clinician diagnosis of obesity or body mass index–based obesity; calculated only in 7603 children older than 2 years.

[g] Including conditions such as fetal alcohol syndrome, Ehlers-Danlos Syndrome, achondroplasia, and chromosomal abnormalities causing malformations.

[h] Pairwise differences between all groups were statistically significant at the P=0.05 level using Dunn's test for multiple comparisons.

Prevalence was calculated based on non-missing data. Abbreviations: MIS-C = multisystem inflammatory syndrome in children. ICU = intensive care unit. ARDS = acute respiratory distress syndrome. BNP = B-type natriuretic peptide. NT-proBNP = N-terminal prohormone of brain natriuretic peptide.

© 2025 Ma KC et al. *JAMA Network Open*.

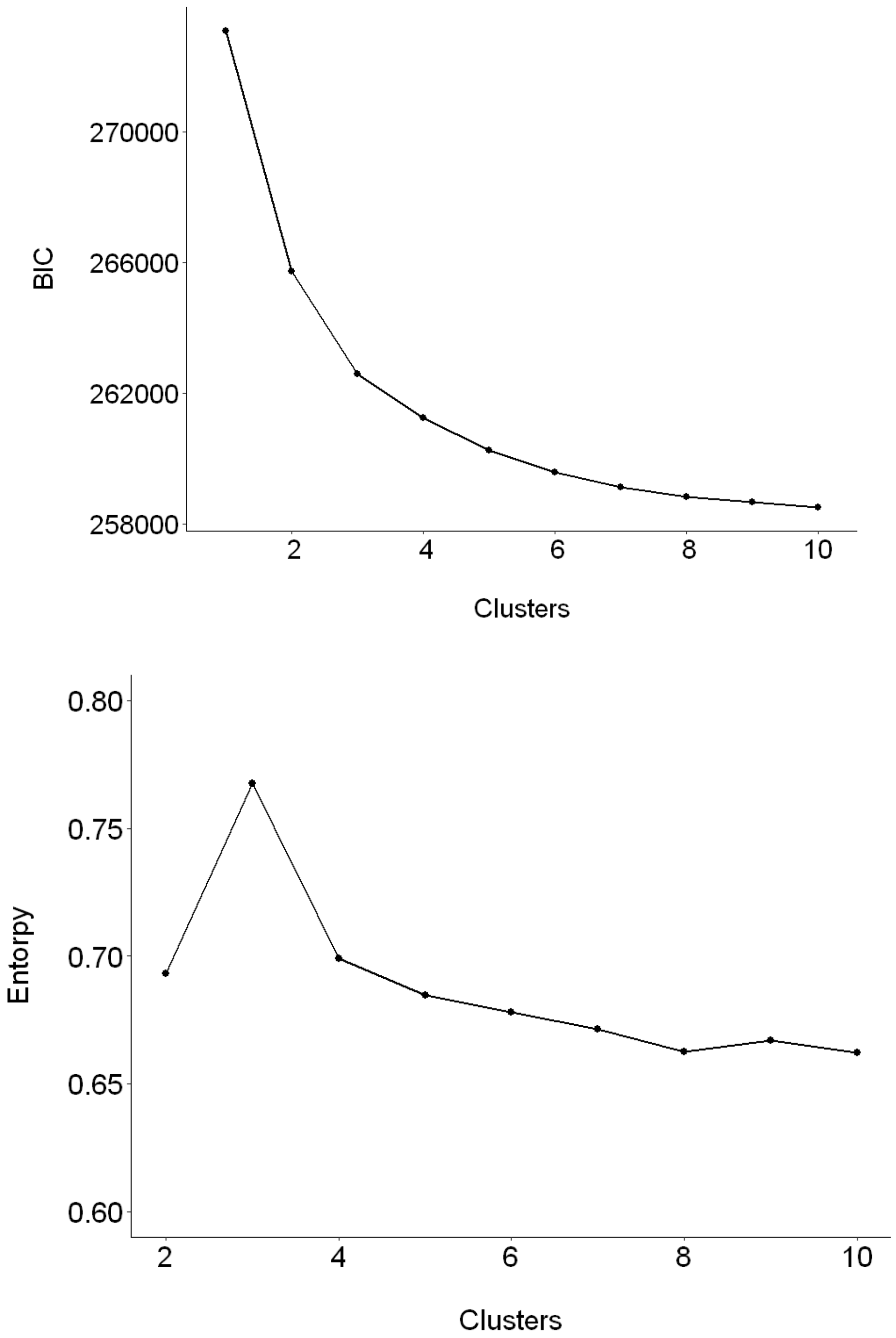

eFigure 1. Bayesian Information Criterion (BIC) values (top) and entropy (bottom) versus number of clusters. We calculated the Bayesian Information Criterion (BIC) for clusters of size one to ten. BIC continued to decrease for each additional class added at least up until ten clusters. Decreases in BIC began to diminish beginning with clusters of size three to five. We calculated relative entropy, which is a measure of cluster distinctiveness ranging from 0 to 1 with higher values indicating greater separation between clusters [1], for clusters of size two to ten. An LCA solution with three clusters had the highest entropy (0.77).

© 2025 Ma KC et al. *JAMA Network Open*.

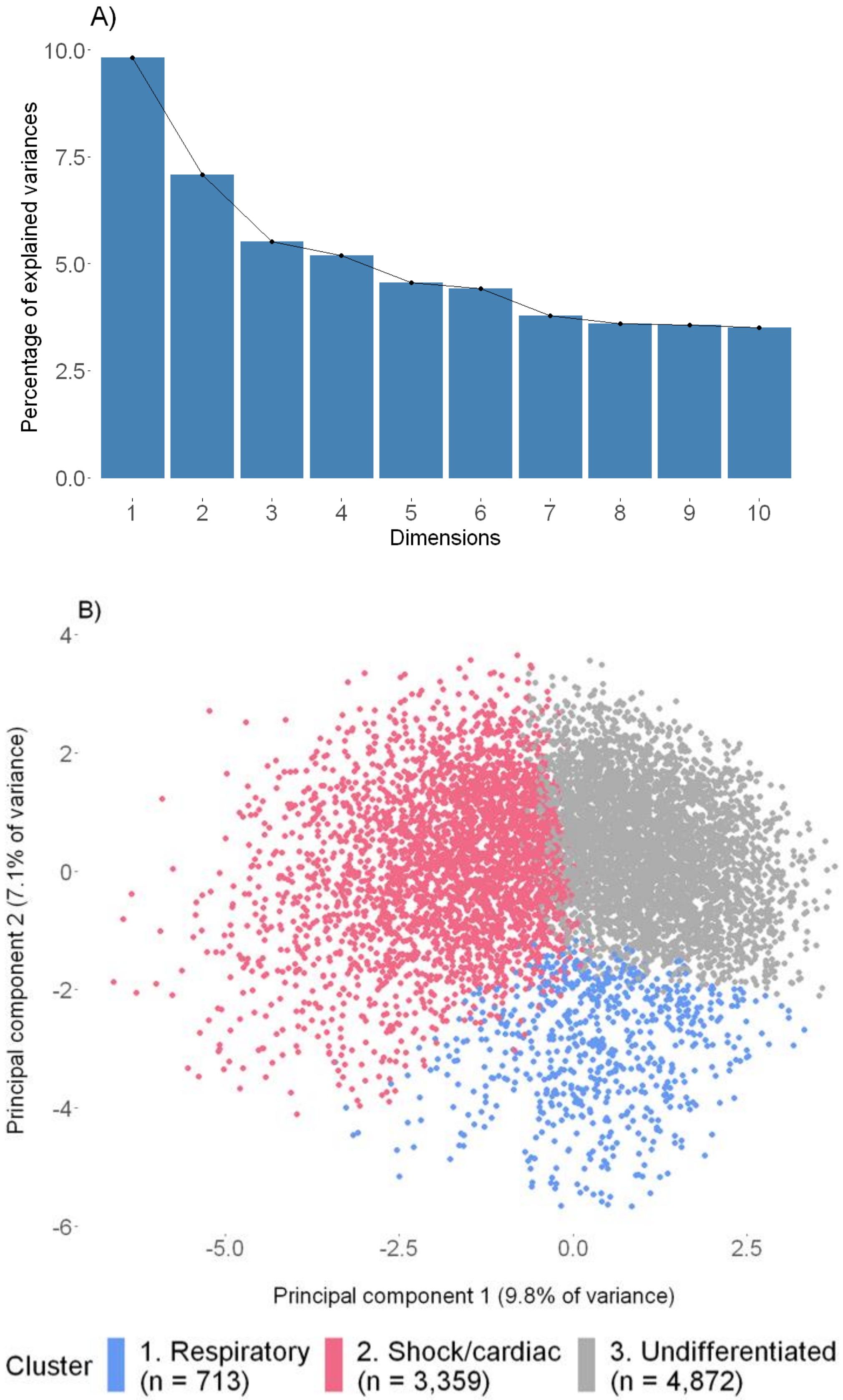

eFigure 2. Principal component analysis (PCA) visualizations of variance explained by principal components (a) and MIS-C cases by latent class analysis-inferred cluster (b). PCA is a variable reduction approach intended to simplify complex datasets into a smaller number of variables (i.e., principal components)

© 2025 Ma KC et al. *JAMA Network Open*.

that contribute the most variance [2]. PCA indicated visual separation in a plot of MIS-C cases along principal components one (x-axis) and two (y-axis) between the three clusters inferred using LCA. Principal components one and two explain only 17% of the total variance, and therefore are a limited representation of the full variation in MIS-C clinical phenotypes. Principal component one correlated most strongly with shock, elevated troponin, and acute kidney injury, and principal component two correlated most strongly with conjunctival injection, pneumonia, and rash.

© 2025 Ma KC et al. *JAMA Network Open*.

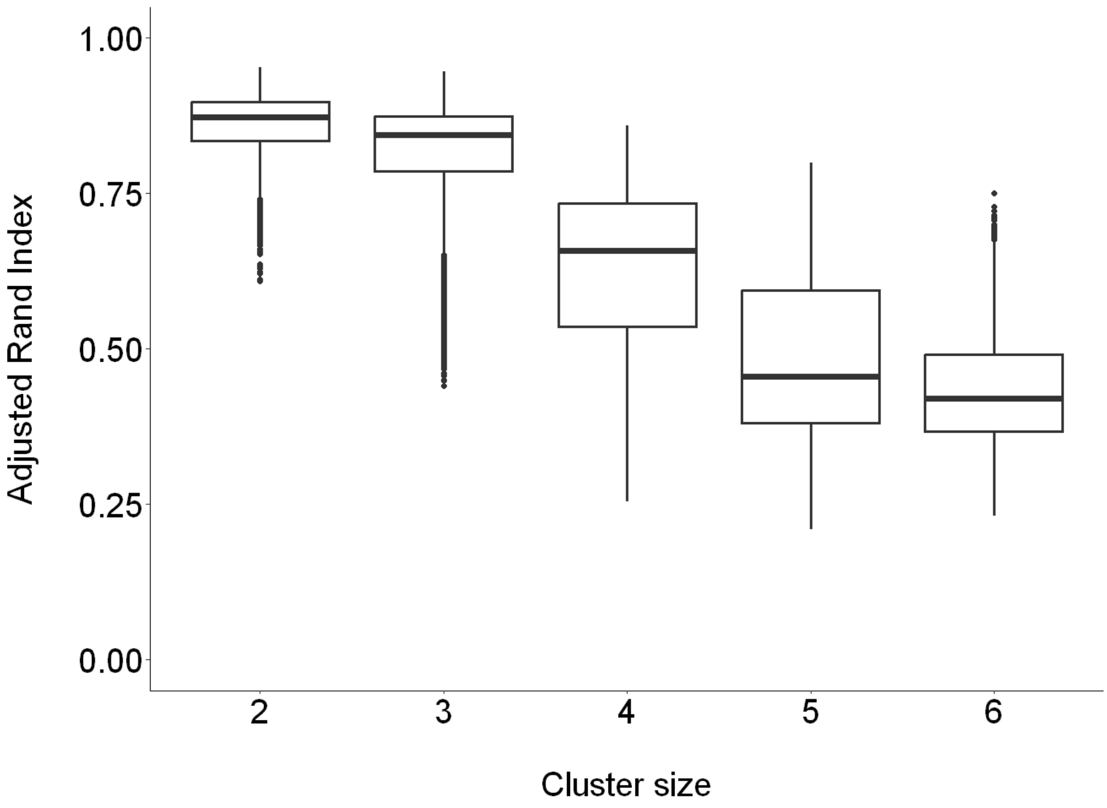

eFigure 3. Consistency of latent class analysis-inferred clusters estimated from sub-sampled datasets. We evaluated how consistent clusters were when subsampling data, preferring solutions with cluster sizes that were less sensitive to changes in the input data, as our case patients come from voluntary surveillance and thus are likely a sample of all MIS-C cases in the U.S. We randomly subsampled n=2000 cases from the dataset 100 times and ran latent class analysis varying the number of clusters from 2 to 6. For each value of cluster size, we used the subsampled LCA models to predict class membership for the full dataset and computed Adjusted Rand Indices, a measure of similarity between two clustering assignments with 1 indicating identical results, for each pair of subsampled models. We observed that LCA solutions with cluster sizes of two and three produced the most consistent estimates, as measured by higher Rand Index values, when the input data were varied in this manner.

© 2025 Ma KC et al. *JAMA Network Open*.

eAppendix. Multisystem inflammatory syndrome associated with COVID-19 2020 case report form

U.S. DEPARTMENT OF
HEALTH AND HUMAN SERVICES
CENTERS FOR DISEASE CONTROL
AND PREVENTION
ATLANTA, GA 30329

Multisystem Inflammatory Syndrome Associated with COVID-19 Case Report Form

MIS ID *(REQUIRED)*: ______________________ **Health Department ID:** ____________________

NNDSS ID *(local_record_id/case id)*: ____________ **Tools for CRF data submission to supplement NNDSS case notification/data:** ○ DCIPHER ○ RedCap

Abstractor name: ______________________ **NCOV ID** *(if available)*: ________________ **Date of abstraction:** ____________

SECTION 1 – INCLUSION CRITERIA

1.1 ☐ Age <21, AND

1.2 ☐ Fever >38.0°C for ≥24 hours, or report of subjective fever lasting ≥24 hours, AND

1.3 ☐ Laboratory markers of inflammation (including, but not limited to one or more; an elevated C-reactive protein (CRP), erythrocyte sedimentation rate (ESR), fibrinogen, procalcitonin, d-dimer, ferritin, lactic acid dehydrogenase (LDH), or interleukin 6 (IL-6), elevated neutrophils, reduced lymphocytes and low albumin, AND

1.4 ☐ Evidence of clinically severe illness requiring hospitalization, with multisystem (≥2) organ involvement *(check all applicable below)*: AND
- **1.4.1** ☐ Cardiac (e.g. shock, elevated troponin, BNP, abnormal echocardiogram, arrhythmia)
- **1.4.2** ☐ Renal (e.g. acute kidney injury or renal failure)
- **1.4.3** ☐ Respiratory (e.g. pneumonia, ARDS, pulmonary embolism)
- **1.4.4** ☐ Hematologic (e.g. elevated D-dimers, thrombophilia, or thrombocytopenia)
- **1.4.5** ☐ Gastrointestinal (e.g. elevated bilirubin, elevated liver enzymes, or diarrhea)
- **1.4.6** ☐ Dermatologic, (e.g. rash, mucocutaneous lesions)
- **1.4.7** ☐ Neurological, (e.g. CVA, aseptic meningitis, encephalopathy)

1.5 ☐ No alternative plausible diagnosis; AND

1.6 ☐ Positive for current or recent SARS-COV-2 infection by (check all applicable below): OR
- **1.6.1** ☐ RT-PCR
- **1.6.2** ☐ Serology
- **1.6.3** ☐ Antigen test

1.7 ☐ COVID-19 exposure within the 4 weeks prior to the onset of symptoms
- **1.7.1** If yes, date of first exposure within the 4 weeks prior : *(MM/DD/YYYY)*: ____________ ☐ Unknown

SECTION 2 – PATIENT DEMOGRAPHICS

2.1 **State of Residence:** ______________________

2.2 **Patient zip code**/postal code *(primary residence)*: ________

2.3 **Date of birth** *(MM/DD/YYYY)*: ____________

2.4 **Sex:** ○ Male ○ Female

2.5 **Ethnicity:** ○ Hispanic or Latino ○ Not Hispanic or Latino ○ Refused or Unknown

2.6 **Race** *(mark all that apply, selecting more than one option as necessary)*:
- **2.6.1** ☐ White
- **2.6.2** ☐ Black or African American
- **2.6.3** ☐ American Indian
- **2.6.4** ☐ Alaska Native or Aboriginal Canadian
- **2.6.5** ☐ Native Hawaiian
- **2.6.6** ☐ Other Pacific Islander
- **2.6.7** ☐ Asian
- **2.6.8** ☐ Other
- **2.6.9** ☐ Refused or Don't know

2.7 **Height:** ________ inches

2.8 **Weight:** ________ lbs

2.9 **BMI:** ________

Comorbidities:

**2.10.1**	Immunosuppressive disorder/malignancy	○ Yes	○ No
**2.10.2**	Obesity	○ Yes	○ No
**2. 10.3**	Type 1 diabetes	○ Yes	○ No
**2. 10.4**	Type 2 diabetes	○ Yes	○ No
**2. 10.5**	Seizures	○ Yes	○ No
**2. 10.6**	Congenital heart disease	○ Yes	○ No
**2. 10.7**	Sickle cell disease	○ Yes	○ No
**2. 10.8**	Chronic lung disease	○ Yes	○ No
**2. 10.9**	Other congenital malformations	○ Yes	○ No
**2. 10.10**	Other *(specify)*: ______________________		

2.11 Hospital admission date *(MM/DD/YYYY)*: ____________
- **2.11.1** Number of days in the hospital: ________

2.12 If admitted to the ICU, admission date *(MM/DD/YYYY)*: ____________
- **2.12.1** Number of days in the ICU: ________

2.13 **Patient outcome:** ○ Died ○ Discharged ○ Still admitted
- **2.13.2** Hospital discharge or death date *(MM/DD/YYYY)*: ____________

© 2025 Ma KC et al. *JAMA Network Open*.

SECTION 3 – CLINICAL SIGNS AND SYMPTOMS

3.1 Did the patient have preceding COVID-like illness? ○ Yes ○ No

3.1.1 Date of symptom onset *(MM/DD/YYYY)*: _______________

3.2 Date of symptom onset of MIS *(MM/DD/YYYY)*: _______________

3.3. Fever ≥ 38.0°C: ○ Yes ○ No

3.3.1 Date of fever onset *(MM/DD/YYYY)*: _______________

3.3.2 Highest Temperature: ______ °C

3.3.3 Number of days febrile: ______

Signs and symptoms ***during present illness***

3.4.1 Cardiac

3.4.1.1	Shock	○ Yes	○ No
3.4.1.2	Elevated troponin	○ Yes	○ No
3.4.1.3	Elevated BNP or NT-proBNP	○ Yes	○ No

3.4.2 Renal

3.4.2.1	Acute kidney injury	○ Yes	○ No
3.4.2.2	Renal failure	○ Yes	○ No

3.4.3 Respiratory

3.4.3.1	Cough	○ Yes	○ No
3.4.3.2	Shortness of breath	○ Yes	○ No
3.4.3.3	Chest pain/tightness	○ Yes	○ No
3.4.3.4	Pneumonia	○ Yes	○ No
3.4.3.5	ARDS	○ Yes	○ No
3.4.3.6	Pulmonary embolism	○ Yes	○ No

3.4.4 Hematologic

3.4.4.1	Elevated D-dimers	○ Yes	○ No
3.4.4.2	Thrombophilia	○ Yes	○ No
3.4.4.3	Thrombocytopenia	○ Yes	○ No

3.4.5 Gastrointestinal

3.4.5.1	Abdominal pain	○ Yes	○ No
3.4.5.2	Vomiting	○ Yes	○ No
3.4.5.3	Diarrhea	○ Yes	○ No
3.4.5.4	Elevated bilirubin	○ Yes	○ No
3.4.5.5	Elevated liver enzymes	○ Yes	○ No

3.4.6 Dermatologic

3.4.6.1	Rash	○ Yes	○ No
3.4.6.2	Mucocutaneous lesions	○ Yes	○ No

3.4.7 Neurological

3.4.7.1	Headache	○ Yes	○ No
3.4.7.2	Altered mental state	○ Yes	○ No
3.4.7.3	Syncope/near syncope	○ Yes	○ No
3.4.7.5	Meningitis	○ Yes	○ No
3.4.7.6	Encephalopathy	○ Yes	○ No

3.4.8 Other

3.4.8.1	Neck pain	○ Yes	○ No
3.4.8.2	Myalgia	○ Yes	○ No
3.4.8.3	Conjunctival injection	○ Yes	○ No
3.4.8.4	Periorbital edema	○ Yes	○ No
3.4.8.5	Cervical lymphadenopathy >1.5 cm diameter	○ Yes	○ No

SECTION 4 – COMPLICATIONS

4.1	**Arrhythmia**	○ Yes	○ No
	If yes:		
4.1.1	Ventricular arrhythmia:	○ Yes	○ No
4.1.2	Supraventricular arrhythmia:	○ Yes	○ No
4.1.3	Other arrhythmia *(specify)*: ____________________	○ Yes	○ No
4.2	Congestive heart failure	○ Yes	○ No
4.3	Myocarditis	○ Yes	○ No
4.4	Pericarditis	○ Yes	○ No
4.5	Liver failure	○ Yes	○ No
4.6	Deep vein thrombosis or PE	○ Yes	○ No
4.7	ARDS	○ Yes	○ No
4.8	Pneumonia	○ Yes	○ No
4.9	CVA or stroke	○ Yes	○ No
4.10	Encephalitis or aseptic meningitis	○ Yes	○ No
4.11	Shock	○ Yes	○ No
4.12	Hypotension	○ Yes	○ No

SECTION 5 – TREATMENTS

5.1	Low flow nasal cannula	○ Yes	○ No
5.2	High flow nasal cannula	○ Yes	○ No
5.3	Non-invasive ventilation	○ Yes	○ No
5.4	Intubation	○ Yes	○ No
5.5	Mechanical ventilation	○ Yes	○ No
5.6	ECMO	○ Yes	○ No
5.7	Vasoactive medications (e.g. epinephrine, milrinone, norepinephrine, or vasopressin) *(specify)*: ____________________	○ Yes	○ No
5.8	Steroids	○ Yes	○ No
5.9	Immune modulators (e.g. anakinra, tocilizumab) *(specify)*: ____________________	○ Yes	○ No
5.10	Antiplatelets (e.g. aspirin, clopidogrel) *(specify)*: ____________________	○ Yes	○ No
5.11	Anticoagulation (e.g. heparin, enoxaparin, warfarin) *(specify)*: ____________________	○ Yes	○ No
5.12	Dialysis	○ Yes	○ No
5.13	First IVIG	○ Yes	○ No
5.14	Second IVIG	○ Yes	○ No

© 2025 Ma KC et al. *JAMA Network Open*.

SECTION 6 – STUDIES

6.1 Blood Test Results

**6.1.1**	Fibrinogen	Highest value: ______	units: ______	○ Low	○ Normal	○ High
**6.1.2**	CRP	Highest value: ______	units: ______	○ Low	○ Normal	○ High
**6.1.3**	Ferritin	Highest value: ______	units: ______	○ Low	○ Normal	○ High
**6.1.4**	Troponin	Highest value: ______	units: ______	○ Low	○ Normal	○ High
**6.1.5**	BNP	Highest value: ______	units: ______	○ Low	○ Normal	○ High
**6.1.6**	NT-proBNP	Highest value: ______	units: ______	○ Low	○ Normal	○ High
**6.1.7**	D-dimer	Highest value: ______	units: ______	○ Low	○ Normal	○ High
**6.1.8**	IL-6	Highest value: ______	units: ______	○ Low	○ Normal	○ High

**6.1.9**	Serum White blood count	Highest value: ______	Lowest value: ______	units: ______
**6.1.10**	Platelets	Highest value: ______	Lowest value: ______	units: ______
**6.1.11**	Neutrophils	Highest value: ______	Lowest value: ______	units: ______
**6.1.12**	Lymphocytes	Highest value: ______	Lowest value: ______	units: ______
**6.1.13**	Bands	Highest value: ______	Lowest value: ______	units: ______

6.2 CSF Studies

**6.2.1**	White blood count	Highest value: ______	Lowest value: ______	units: ______
**6.2.2**	Protein	Highest value: ______	Lowest value: ______	units: ______
**6.2.3**	Glucose	Highest value: ______	Lowest value: ______	units: ______

6.3 Urinalysis

**6.3.1**	Urine White blood count	Highest value: ______	Lowest value: ______	units: ______

6.4 Echocardiogram (check if seen on ANY echocardiogram)

6.4.1 ☐ Not done
6.4.2 ☐ Normal results
6.4.3 ☐ Coronary artery aneurysms
  6.4.3.1 Max coronary artery Z-score: ______
6.4.4 ☐ Coronary artery dilatation
6.4.5 ☐ Cardiac dysfunction (decreased function), specify type:
  6.4.5.1 ☐ left ventricular dysfunction
  6.4.5.2 ☐ right ventricular dysfunction
6.4.6 ☐ Pericardial effusion
6.4.7 ☐ Pleural effusion
6.4.8 ☐ Mitral regurgitation, specify type: ○ mild ○ moderate ○ severe
6.4.9 ☐ Other *(specify)*: ______

6.5 Date of first test showing coronary artery aneurysm or dilatation *(MM/DD/YYYY)*: ______

6.6 Abdominal imaging ☐ Ultrasound ☐ CT ☐ Not done

6.6.1 ☐ Normal
6.6.2 ☐ Mesenteric lymphadenopathy
6.6.3 ☐ Free fluid
6.6.4 ☐ Other *(specify)*: ______

6.7 Chest imaging ☐ Chest x-ray ☐ CT ☐ Not done

6.7.1 ☐ Normal
6.7.2 ☐ Pneumonia
6.7.3 ☐ Atelectasis
6.7.4 ☐ Pleural effusion
6.7.5 ☐ Other *(specify)*: ______

SARS-COV-2 testing

6.8 RT-PCR: ○ Positive ○ Negative ○ Not done
6.8.1 If performed, date *(MM/DD/YYYY)*: ______

6.9 Antigen: ○ Positive ○ Negative ○ Not done
6.9.1 If performed, date *(MM/DD/YYYY)*: ______

6.10 IgG: ○ Positive ○ Negative ○ Not done
6.10.1 If performed, date *(MM/DD/YYYY)*: ______

6.11 IgM: ○ Positive ○ Negative ○ Not done
6.11.1 If performed, date *(MM/DD/YYYY)*: ______

6.12 IgA: ○ Positive ○ Negative ○ Not done
6.12.1 If performed, date *(MM/DD/YYYY)*: ______

© 2025 Ma KC et al. *JAMA Network Open*.

SECTION 7 COVID-19 VACCINE INFORMATION

7.1 **Has the patient received a COVID-19 vaccine?** ○ Yes ○ No ○ Unknown

7.2 **If yes, how many doses?** ○ 1 dose ○ 2 doses ○ Unknown

7.2.1 Date dose 1 received *(MM/DD/YYYY)*: ______________

7.2.2 Date dose 2 received *(MM/DD/YYYY)*: ______________

7.3 **COVID-19 Vaccine manufacturer** ○ Pfizer ○ Moderna ○ Johnson & Johnson/Janssen

○ Other, *(specify)*: ____________________________________ ○ Unknown

RESET FORM

© 2025 Ma KC et al. *JAMA Network Open*.

eReferences

1. Sinha P, Calfee CS, Delucchi KL. Practitioner's Guide to Latent Class Analysis: Methodological Considerations and Common Pitfalls. Crit Care Med **2021**; 49:e63–e79.

2. Greenacre M, Groenen PJF, Hastie T, D'Enza AI, Markos A, Tuzhilina E. Principal component analysis. Nature Reviews Methods Primers **2022**; 2:1–21.

3. Fop M, Smart KM, Murphy TB. Variable selection for latent class analysis with application to low back pain diagnosis. Ann Appl Stat **2017**; 11:2080–2110.

© 2025 Ma KC et al. *JAMA Network Open*.
