# Supplementary material for: Phenotypic Classification of Multisystem Inflammatory Syndrome in Children Using Latent Class Analysis
Source: JAMA Netw Open. 2025 Jan 28;8(1):e2456272. doi: 10.1001/jamanetworkopen.2024.56272 (PMC11775748; doi:10.1001/jamanetworkopen.2024.56272)
Supplement: Supplement 2. — Data Sharing Statement [file jamanetwopen-e2456272-s002.pdf]

Data Sharing Statement

Ma. Phenotypic Classification of Multisystem Inflammatory Syndrome in Children Using Latent Class Analysis. *JAMA Netw Open*. Published January 28, 2025. doi:10.1001/jamanetworkopen.2024.56272

Data
Data available: Yes
Data types: Deidentified participant data
How to access data: Data available: Yes Data types: Deidentified participant data in limited dataset How to access data: only upon formal request When available: With publication
When available: With publication

Supporting Documents
Document types: None

Additional Information
Who can access the data: Who can access the data: researchers whose proposed use of the data has been approved
Types of analyses: Types of analyses: For a specified purpose
Mechanisms of data availability: Mechanisms of data availability: with a signed data use agreement
